# Unveiling the Molecular Mechanism of 6PPD and 6PPD-Q in Lipid-Metabolism-Related Diseases Through Network Toxicology and Experimental Validation

**DOI:** 10.3390/ijms27156712

**Published:** 2026-07-27

**Authors:** Ze Li, Yuyang Luo, Jianan Zhao, Siyi Wang, Yixuan Zhang

**Affiliations:** 1 Department of Physiology, School of Basic Medical Science, Central South University, Changsha 410011, China; 2 Graduate School, Shanghai University of Traditional Chinese Medicine, Shanghai 201203, China; 3 Xiangya School of Medicine, Central South University, Changsha 410011, China

**Keywords:** 6PPD, 6PPD-quinone, environmental factors, network toxicology, lipid-metabolism disorders, molecular docking

## Abstract

6PPD and its ozonation product 6PPD-quinone (6PPD-Q) are ubiquitous tire-derived pollutants linked to environmental and potential human health risks. This study systematically investigated their mechanisms in lipid-metabolism-related diseases (atherosclerosis, type 2 diabetes, and nonalcoholic fatty liver disease) through network toxicology, transcriptomic validation, molecular docking, and experimental models. Targets of 6PPD and 6PPD-Q were predicted using multiple databases and intersected with disease-associated genes. Protein–protein interaction networks, hub gene screening, GO/KEGG enrichment, and GEO transcriptomic datasets identified key shared core targets, including PTGS2, MMP9, CXCL8 (for 6PPD), MAPK14, and PTGS2 (for 6PPD-Q). Molecular docking suggested potential strong binding affinities. Integrative analysis highlighted convergence on oxidative stress, inflammation, lipid dysregulation, and MAPK signaling. In vivo, 40-day exposure to 6PPD and 6PPD-Q in C57BL/6 mice induced hepatic steatosis, elevated serum TC, LDL-C, and HDL-C, upregulated inflammatory cytokines (TNF-α, IL1B, IL6, and IFNG), and core targets. In vitro, both compounds caused dose-dependent cytotoxicity, ROS accumulation, glutathione redox imbalance, and pro-inflammatory activation. These findings suggest that 6PPD and 6PPD-Q may contribute to lipid-metabolism-related toxic responses through shared and distinct processes involving oxidative stress, inflammatory activation, and lipid dysregulation. These results provide preliminary mechanistic insights into their metabolic toxicity and support further experimental evaluation for environmental health risk assessment.

## 1. Introduction

The growth of globalization and the spread of urban transportation have greatly increased the size of the automobile industry. As a result, tire production and use have continued to rise over time. Tires are necessary for vehicles to run. To reduce rubber aging caused by heat and ozone and to make tires last longer, p-phenylenediamine antioxidants are often added during tire production. One of the most common additives is N-(1,3-dimethylbutyl)-N’-phenyl-p-phenylenediamine (6PPD), which is widely used in the tire industry [1,2]. During driving, constant friction between tires and roads releases tire wear particles that contain 6PPD into road dust, air particles, soil, and urban runoff. When ozone is present in the environment, 6PPD can change into a more reactive and more toxic product called 6PPD-quinone (6PPD-Q or 6PPDQ) [2,3,4]. In recent years, both 6PPD and 6PPD-Q have often been found in road dust, airborne particles, stormwater runoff, surface water, and soil. This shows that they are now common traffic-related pollutants from tires [2,3].

After 6PPD-Q was found to be the main chemical causing the death of coho salmon in urban runoff, people began to pay more attention to the environmental pollution and health risks caused by tire-related pollutants [4]. As human exposure studies have developed, 6PPD, 6PPD-Q, and other related PPD-Qs have also been found in human urine, serum, and breast milk. This suggests that these pollutants are not only present in the outside environment but have also entered the human body through exposure [5,6,7]. More toxicology studies have shown that the harmful effects of 6PPD and 6PPD-Q are not limited to aquatic organisms. They can also cause damage to many organs in mammals, including the liver, lung, reproductive system, metabolic organs, and cardiovascular tissues [8].

Lipid-metabolism disorder is an important basis for many chronic non-communicable diseases. Atherosclerosis, diabetes, and nonalcoholic fatty liver disease (NAFLD) have different clinical features and affect different main organs. Even so, they are all closely linked to an imbalance between lipid production and lipid breakdown, long-term low-grade inflammation, and oxidative stress [9,10]. NAFLD is one of the most common chronic liver diseases, and it can develop into steatohepatitis and even cirrhosis [10]. Diabetes, especially type 2 diabetes, is marked by insulin resistance and abnormal glucose and lipid metabolism [9]. Atherosclerosis is closely linked to endothelial injury, lipid buildup, inflammatory activation, and remodeling of the blood vessel wall [11]. For this reason, environmental pollutants that cause oxidative stress, inflammatory imbalance, and disturbed lipid homeostasis may help start and worsen these diseases.

In line with this disease’s background, several experimental studies have suggested that 6PPD and 6PPD-Q may strongly affect lipid metabolism. In Caenorhabditis elegans, 6PPD-Q can raise triglyceride levels, enlarge lipid droplets, and increase fatty acid synthesis, and this effect can even continue across generations [12]. In mice, both 6PPD and 6PPD-Q can cause lipid buildup in the liver, and low-dose exposure to 6PPD-Q has been shown to produce a fatty liver phenotype by increasing fatty acid biosynthesis [13]. Multi-omics studies also suggest that 6PPD and 6PPD-Q may promote NAFLD-related changes by disturbing the balance of the gut-liver axis [14]. Taken together, these findings suggest that tire-derived pollutants may interfere with lipid metabolism and potentially contribute to pathological processes associated with lipid-metabolism-related diseases.

In addition to liver lipid buildup, the link between 6PPD and 6PPD-Q and diabetes-related changes has also received more attention. Earlier studies showed that oral exposure to 6PPD-Q can cause weight gain, higher fasting blood glucose, insulin resistance, and changes like metabolic syndrome. These effects were linked to inhibition of the PI3K–AKT signaling pathway [15]. In human studies, higher serum 6PPD-Q levels were linked to abnormal increases in triglycerides and a greater risk of liver injury [8]. In contrast, there is still no direct evidence linking 6PPD and 6PPD-Q with atherosclerosis. However, current studies have shown that these compounds can cause abnormal vascular permeability, endothelial injury, oxidative stress, and heart dysfunction [16,17]. These processes are very similar to the main events in atherosclerosis. This suggests that 6PPD and 6PPD-Q may be involved in cardiovascular disease through a process in which endothelial injury and inflammatory imbalance make lipid buildup more likely. Even so, there is still no clear and direct evidence showing whether they can directly promote plaque formation, change plaque stability, or cause typical atherosclerotic changes.

Although the lipid metabolic toxicity of 6PPD and 6PPD-Q has been studied in different models and from different angles, several important limits still exist. First, most current studies focus on only one organ or one pathway, so it is hard to explain the multi-target, multi-pathway, and multi-disease features of 6PPD and 6PPD-Q at the whole-system level. Second, although 6PPD and 6PPD-Q clearly have a precursor–metabolite relationship, their starting molecules, main toxic targets, and key signaling pathways have not been compared in a systematic way. Third, although atherosclerosis, diabetes, and NAFLD all share lipid-metabolism disorder as a common pathological basis, it is still unknown whether pollutant-responsive networks contain shared targets or disease-specific modules.

Beyond their toxicological effects in experimental models, 6PPD and 6PPD-Q may also represent emerging environmental determinants of chronic metabolic health. Tire wear particles are continuously released from road traffic and have become ubiquitous contaminants in urban air, road dust, stormwater runoff, and other environmental media. Consequently, human exposure may occur through multiple pathways, including inhalation of airborne particles, ingestion of contaminated dust, and dermal contact. Unlike acute toxicants, chronic exposure to low levels of environmental pollutants may contribute to long-term biological stress characterized by oxidative imbalance, low-grade inflammation, and metabolic dysfunction. Because atherosclerosis, type 2 diabetes, and NAFLD are among the most prevalent non-communicable diseases worldwide, identifying environmental factors that may influence their development has important implications for environmental health surveillance, risk assessment, and public health prevention strategies.

Network toxicology combines information on compound targets, disease genes, protein–protein interactions, and pathway enrichment, so it is very useful for studying the multi-target, weak-effect, and long-term exposure features of environmental pollutants. When combined with molecular docking, it can also help identify key nodes and check possible binding patterns [18,19]. Based on this method, the present study used network toxicology, protein–protein interaction analysis, enrichment analysis, molecular docking, and experimental validation to systematically describe the possible action networks of 6PPD and 6PPD-Q in diseases linked to lipid-metabolism disorders. We focused on three representative metabolic diseases: atherosclerosis, type 2 diabetes, and NAFLD. By comparing the action targets, key signaling pathways, and core network modules of the two compounds across these diseases, we aimed to build a mechanistic framework for the metabolic toxicity caused by 6PPD and 6PPD-Q. We also aimed to provide a theoretical basis for understanding the metabolic health risks of this group of tire-derived pollutants, finding possible biomarkers, and supporting future risk assessment.

## 2. Results

### 2.1. Toxicological Profiling of 6PPD and 6PPD-Q

To evaluate the potential human health risks of 6PPD and 6PPD-Q, their toxicological profiles were predicted using the ADMETlab 3.0 platform (Table 1). Both compounds showed high predicted probabilities for several toxicity endpoints, including genotoxicity and skin sensitization. Consistent with the focus of this study on lipid-metabolism-related disorders, both pollutants also showed predicted hepatotoxic potential. 6PPD showed a high predicted risk for drug-induced liver injury (DILI, 0.920) and human hepatotoxicity (0.788), while the predicted probability of human hepatotoxicity was 0.830 for 6PPD-Q. In addition, 6PPD showed higher predicted respiratory and nephrotoxicity risks, whereas 6PPD-Q showed a higher predicted hematotoxicity probability. These results provide a toxicological basis for further investigating their potential metabolic effects.

### 2.2. The Effects of 6PPD on Atherosclerosis

**Figure 1:**
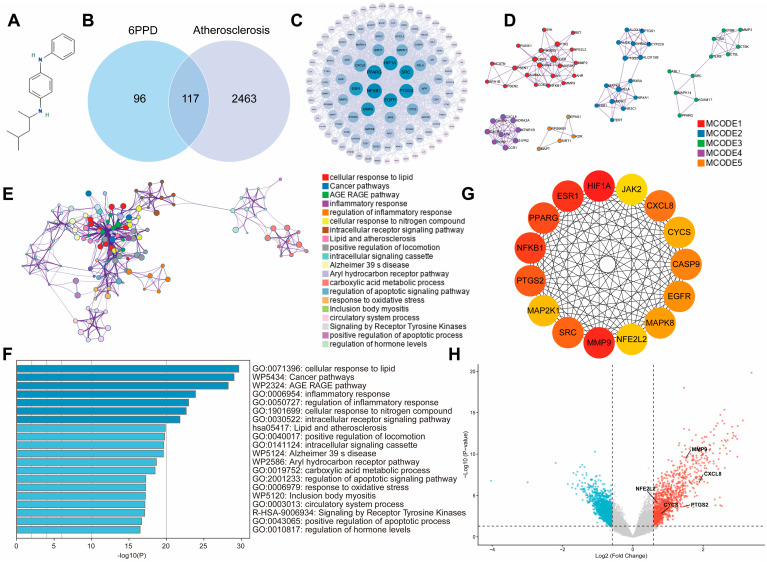
PPI network construction and enrichment analysis of 6PPD-associated targets in atherosclerosis. (**A**) 2D chemical structure of 6PPD derived from its canonical SMILES. (**B**) Venn diagram illustrating the intersection of predicted 6PPD targets and atherosclerosis-associated genes. (**C**) PPI network of the shared targets constructed via the STRING database (confidence score > 0.40). (**D**) Key functional modules extracted from the PPI network using the MCODE algorithm. (**E**) Network visualization of the enriched GO and KEGG pathways for the intersecting targets. (**F**) Bar chart displaying the top significantly enriched functional pathways, ranked by −log10 (*p*). (**G**) The top 15 hub genes prioritized using the MCC method. The color gradient indicates the topological essentiality of the nodes. (**H**) Transcriptomic validation of the identified hub genes using the atherosclerosis clinical dataset GSE66360. The volcano plot highlights the differentially expressed core targets (thresholds: *p* < 0.05 and fold change > 1.5).

To explore the potential molecular targets of 6PPD, its canonical SMILES string was retrieved from the PubChem database and utilized to map its 2D chemical structure (Figure 1A). Potential targets of 6PPD were compiled from the SwissTargetPrediction, TargetNet, ChEMBL, and STITCH databases, yielding a total of 213 candidate genes. Concurrently, 2580 atherosclerosis-related targets were retrieved from the GeneCards database. Venn analysis identified 117 shared targets between 6PPD and atherosclerosis, representing the potential core action network (Figure 1B). These overlapping genes were subsequently imported into the STRING database to construct a PPI network with a minimum confidence score of 0.40 (Figure 1C).

Topological module analysis of the PPI network revealed several highly interconnected clusters. MCODE enrichment demonstrated that these modules were primarily associated with the aryl hydrocarbon receptor pathway (WP2586), serotonergic synapse (hsa04726), arachidonate metabolism (R-HSA-2142753), trafficking and processing of endosomal TLR (R-HSA-1679131), collagen catabolic process (GO:0030574), and degradation of the extracellular matrix (R-HSA-1474228) (Figure 1D; Table S1). These functional profiles indicate that 6PPD may exacerbate atherosclerosis via xenobiotic responses, lipid-related inflammatory mediator metabolism, innate immune signaling, and extracellular matrix remodeling.

Comprehensive gene set enrichment analysis further highlighted significant enrichment in the cellular response to lipid (GO:0071396), inflammatory response (GO:0006954), regulation of inflammatory response (GO:0050727), and lipid and atherosclerosis (hsa05417). This pattern firmly underscores disturbed lipid homeostasis and inflammatory activation as defining pathological features of 6PPD exposure in the context of atherosclerosis. Furthermore, response to oxidative stress (GO:0006979) and regulation of apoptotic signaling pathway (GO:2001233) were also significantly enriched, implicating oxidative injury and apoptosis in endothelial dysfunction and subsequent plaque progression (Figure 1E,F).

To pinpoint critical network nodes, topological ranking via the MCC algorithm prioritized the top-15 candidate hub genes (Figure 1G). Their expression profiles were subsequently validated utilizing the atherosclerosis-related clinical transcriptomic dataset GSE66360. Notably, five core targets exhibited significant transcriptional upregulation, including MMP9, CXCL8, NFE2L2, CYCS, and PTGS2. These substantial transcriptional alterations firmly corroborate their active roles in driving the disease pathology (Figure 1H).

### 2.3. The Effects of 6PPD on Type 2 Diabetes

**Figure 2:**
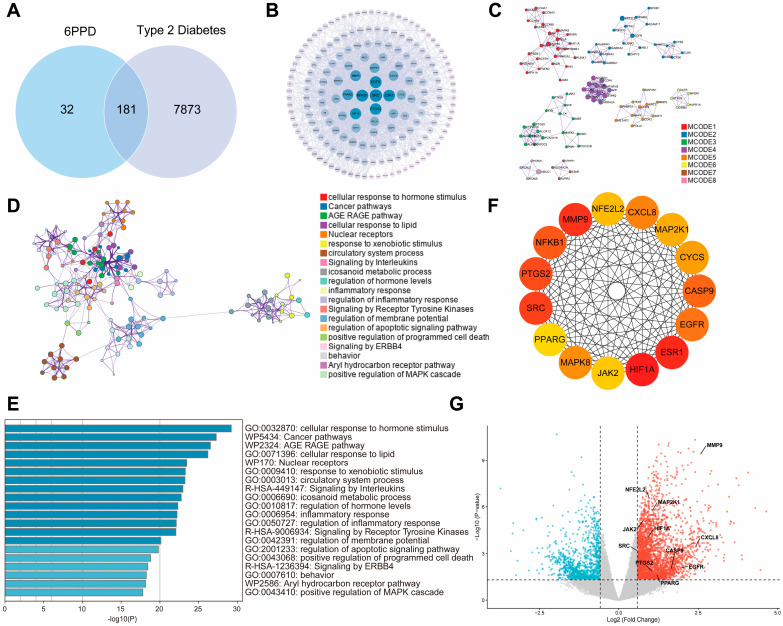
PPI network construction and enrichment analysis of 6PPD-associated targets in type 2 diabetes. (**A**) Venn diagram illustrating the intersection of predicted 6PPD targets and type-2-diabetes-associated genes. (**B**) PPI network of the shared targets constructed via the STRING database (confidence score > 0.40). (**C**) Key functional modules extracted from the PPI network using the MCODE algorithm. (**D**) Network visualization of the enriched GO and KEGG pathways for the intersecting targets. (**E**) Bar chart displaying the top significantly enriched functional pathways, ranked by −log10 (*p*). (**F**) The top 15 hub genes prioritized using the MCC method. The color gradient indicates the topological essentiality of the nodes. (**G**) Transcriptomic validation of the identified hub genes using the type 2 diabetes clinical dataset GSE29231. The volcano plot highlights the differentially expressed core targets (thresholds: *p* < 0.05 and fold change > 1.5).

In the type 2 diabetes analysis, 8054 disease-associated targets were retrieved from GeneCards and compared with the predicted 6PPD target set. This comparison yielded 181 overlapping genes, which were used to generate the corresponding PPI network (Figure 2A,B). MCODE analysis showed that the major network modules were associated with regulated proteolysis of p75NTR (R-HSA-193692), NRIF signals cell death from the nucleus (R-HSA-205043), eicosanoid metabolic process (GO:0006690), and arachidonic acid metabolism (hsa00590) (Figure 2C; Table S1). These results suggest that 6PPD may contribute to diabetes-associated pathological changes by affecting neuroregulatory signaling and metabolic inflammatory responses.

GO/KEGG enrichment further showed that 6PPD-targeted genes in the type 2 diabetes setting were enriched in cellular response to hormone stimulus (GO:0032870), the AGE-RAGE pathway (WP2324), cellular response to lipid (GO:0071396), inflammatory response (GO:0006954), regulation of inflammatory response (GO:0050727), signaling by interleukins (R-HSA-449147), and regulation of apoptotic signaling pathway (GO:2001233). These pathways indicate that 6PPD exposure may disturb hormonal responsiveness, glycation stress, lipid metabolic homeostasis, inflammation, and apoptosis in diabetes, which may severely aggravate islet dysfunction and diabetic complications (Figure 2D,E). Hub-gene screening and validation in GSE29231 identified eleven differentially expressed core targets, including CASP9, CXCL8, EGFR, HIF1A, JAK2, MAP2K1, MMP9, NFE2L2, PPARG, PTGS2, and SRC (Figure 2F,G).

### 2.4. The Effects of 6PPD on NAFLD

**Figure 3:**
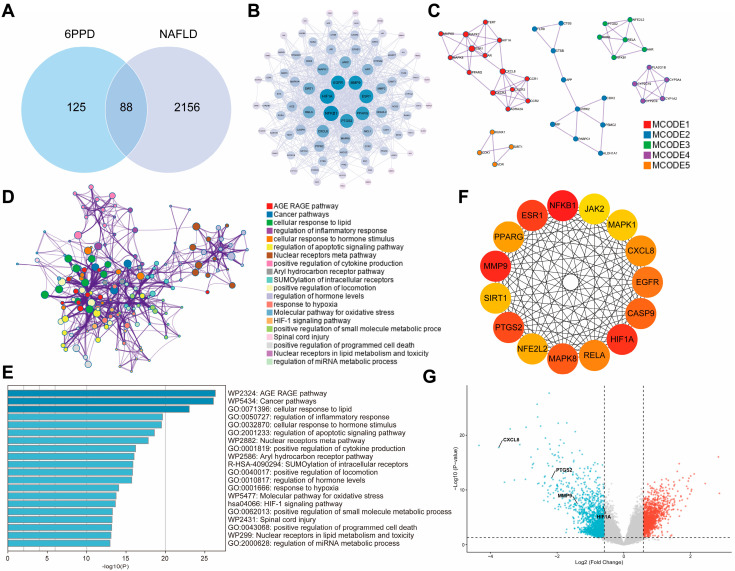
PPI network construction and enrichment analysis of 6PPD-associated targets in NAFLD. (**A**) Venn diagram illustrating the intersection of predicted 6PPD targets and NAFLD-associated genes. (**B**) PPI network of the shared targets constructed via the STRING database (confidence score > 0.40). (**C**) Key functional modules extracted from the PPI network using the MCODE algorithm. (**D**) Network visualization of the enriched GO and KEGG pathways for the intersecting targets. (**E**) Bar chart displaying the top significantly enriched functional pathways, ranked by −log10 (*p*). (**F**) The top 15 hub genes prioritized using the MCC method. The color gradient indicates the topological essentiality of the nodes. (**G**) Transcriptomic validation of the identified hub genes using the NAFLD clinical dataset GSE89632. The volcano plot highlights the differentially expressed core targets (thresholds: *p* < 0.05 and fold change > 1.5).

For the NAFLD-related analysis, 2244 disease-associated targets were collected from GeneCards. Intersecting these genes with the predicted 6PPD targets identified 88 shared genes, which were subsequently used for PPI network construction (Figure 3A,B).

MCODE analysis showed that the core modules were associated with cancer pathways (WP5434), trafficking and processing of endosomal TLR (R-HSA-1679131), the aryl hydrocarbon receptor pathway (WP2586), and linoleic acid metabolism (hsa00591) (Figure 3C; Table S1). This functional profile suggests the involvement of inflammatory and immune activation, xenobiotic responses, and lipid metabolic alterations in 6PPD-induced NAFLD progression. Comprehensive gene set enrichment analysis indicated that the primary enriched terms encompassed the AGE-RAGE pathway (WP2324), cellular response to lipid (GO:0071396), regulation of inflammatory response (GO:0050727), and cellular response to hormone stimulus (GO:0032870). These results strongly link 6PPD exposure to disturbed hepatic lipid metabolism, glycation stress, and hormonal dysregulation. Furthermore, significant enrichment in regulation of apoptotic signaling pathway (GO:2001233), response to hypoxia (GO:0001666), and the HIF-1 signaling pathway (hsa04066) implies that 6PPD may exacerbate hepatocyte injury via severe inflammation, hypoxic stress, and apoptotic cell death, thereby driving NAFLD toward a more progressive state (Figure 3D,E). Hub-gene screening and validation in GSE89632 identified four significant core targets, including CXCL8, HIF1A, MMP9, and PTGS2 (Figure 3F,G).

### 2.5. Molecular Docking of Core Targets Induced by 6PPD in Atherosclerosis, Type 2 Diabetes, and NAFLD

**Figure 4:**
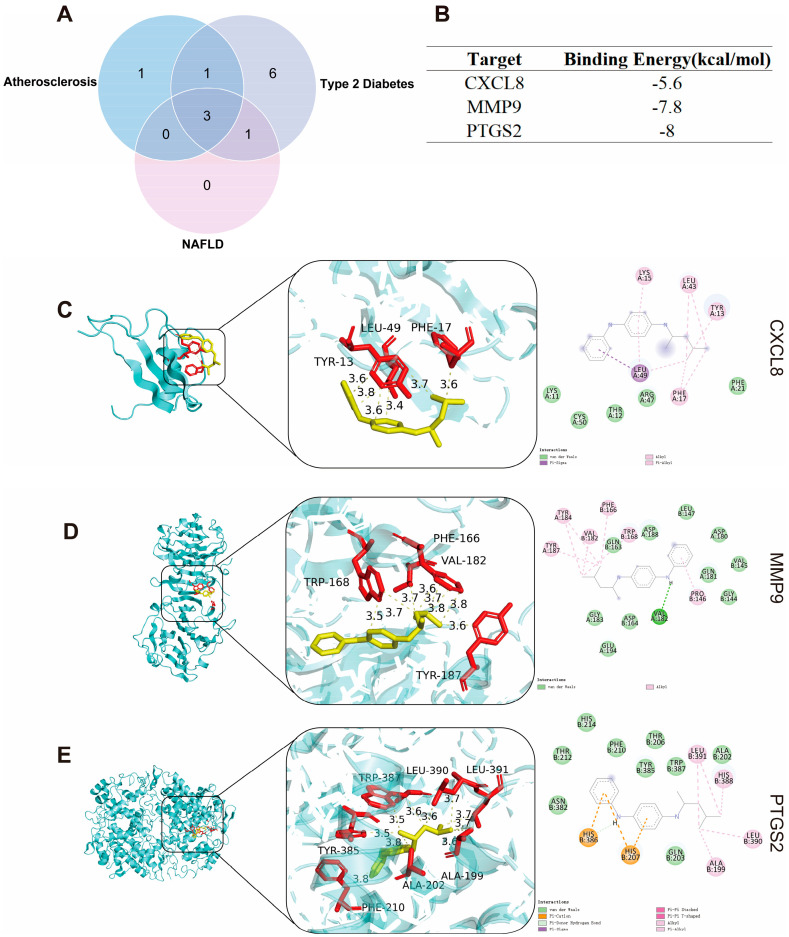
Molecular docking analysis of 6PPD with the shared core targets in lipid-metabolism disorders. (**A**) Venn diagram illustrating the intersection of 6PPD-associated targets with lipid-metabolism disorders, identifying three shared core targets. (**B**) Summary of the binding energies (kcal/mol) between 6PPD and the three shared targets, calculated via AutoDock Vina. (**C**–**E**) Molecular docking simulations demonstrating the optimal binding modes of 6PPD within the active pockets of (**C**) CXCL8, (**D**) MMP9, and (**E**) PTGS2. Each panel displays the 3D protein–ligand complex (left), a magnified 3D view highlighting specific interacting amino acid residues and bond distances (middle), and a 2D interaction diagram detailing the types of intermolecular forces, such as hydrogen bonds and hydrophobic contacts (right).

Venn analysis across the three representative metabolic diseases (atherosclerosis, type 2 diabetes, and NAFLD) identified three universally shared core targets for 6PPD, specifically CXCL8, MMP9, and PTGS2 (Figure 4A). Molecular docking simulations were subsequently employed to evaluate the binding affinities between 6PPD and these shared target proteins (Figure 4B–E). The analyses revealed highly favorable binding configurations, yielding binding energies of −5.6, −7.8, and −8.0 kcal/mol for CXCL8, MMP9, and PTGS2, respectively, indicating potentially spontaneous and highly stable intermolecular interactions. Among them, PTGS2 formed the most stable complex with 6PPD.

Detailed interaction analysis showed that 6PPD adopted favorable docking conformations within the predicted binding regions of CXCL8, MMP9, and PTGS2. These predicted complexes were mainly stabilized by non-covalent interactions, including hydrogen bonds and hydrophobic contacts, with surrounding amino acid residues. Collectively, these docking results indicate potential structural compatibility between 6PPD and these shared inflammatory and metabolic hub proteins, supporting their relevance as common molecular nodes in the 6PPD-associated lipid-metabolism-related disease network (Figure 4C–E).

### 2.6. The Effects of 6PPD-Q on Atherosclerosis

**Figure 5:**
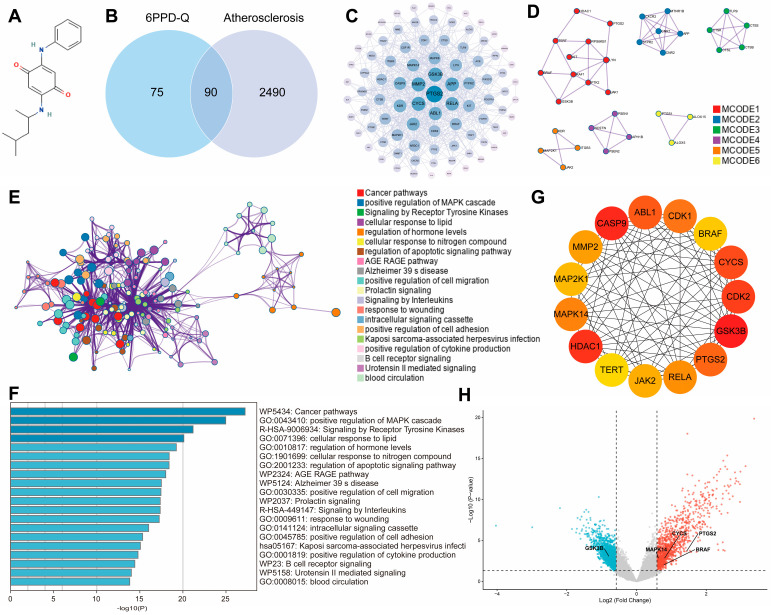
PPI network construction and enrichment analysis of 6PPD-Q-associated targets in atherosclerosis. (**A**) 2D chemical structure of 6PPD-Q derived from its canonical SMILES. (**B**) Venn diagram illustrating the intersection of predicted 6PPD-Q targets and atherosclerosis-associated genes. (**C**) PPI network of the shared targets constructed via the STRING database (confidence score > 0.40). (**D**) Key functional modules extracted from the PPI network using the MCODE algorithm. (**E**) Network visualization of the enriched GO and KEGG pathways for the intersecting targets. (**F**) Bar chart displaying the top significantly enriched functional pathways, ranked by −log10 (*p*). (**G**) The top 15 hub genes prioritized using the MCC method. The color gradient indicates the topological essentiality of the nodes. (**H**) Transcriptomic validation of the identified hub genes using the atherosclerosis clinical dataset GSE66360. The volcano plot highlights the differentially expressed core targets (thresholds: *p* < 0.05 and fold change > 1.5).

For 6PPD-Q, the canonical SMILES string was used to generate its 2D chemical structure (Figure 5A), and 165 candidate targets were compiled from SwissTargetPrediction, TargetNet, ChEMBL, and STITCH. Among these targets, 90 overlapped with atherosclerosis-related genes and were used to construct the 6PPD-Q–atherosclerosis PPI network (Figure 5B,C).

MCODE module analysis highlighted several critical functional clusters (Figure 5D). In atherosclerosis, 6PPD-Q-targeted genes were predominantly enriched in the trafficking and processing of endosomal TLR (R-HSA-1679131) and Toll-like receptor cascades (R-HSA-168898), firmly establishing a link to innate immune recognition and inflammatory signaling cascades. Furthermore, enrichment in class A/1 (rhodopsin-like receptors) (R-HSA-373076) and eicosanoid synthesis (WP167) underscores potential roles in vascular remodeling and lipid-related inflammatory mediator metabolism (Table S2).

Comprehensive ranking analysis revealed marked enrichment in cancer pathways (WP5434), positive regulation of MAPK cascade (GO:0043410), and signaling by receptor tyrosine kinases (R-HSA-9006934), consistent with perturbed vascular cell stress responses driven by enhanced kinase signaling and receptor-mediated signal transduction. Strong enrichment was also observed in cellular response to lipid (GO:0071396), the AGE-RAGE pathway (WP2324), signaling by interleukins (R-HSA-449147), and positive regulation of cell adhesion (GO:0045785). These pathways collectively suggest that 6PPD-Q exacerbates vascular wall injury and atherosclerosis progression via lipid metabolic imbalance, inflammatory activation, and altered endothelial adhesion dynamics (Figure 5E,F). Subsequent transcriptomic validation in GSE66360, visualized by a volcano plot, highlighted BRAF, CYCS, GSK3B, MAPK14, and PTGS2 as differentially expressed hub targets in the 6PPD-Q–atherosclerosis network (Figure 5G,H).

### 2.7. The Effects of 6PPD-Q on Type 2 Diabetes

**Figure 6:**
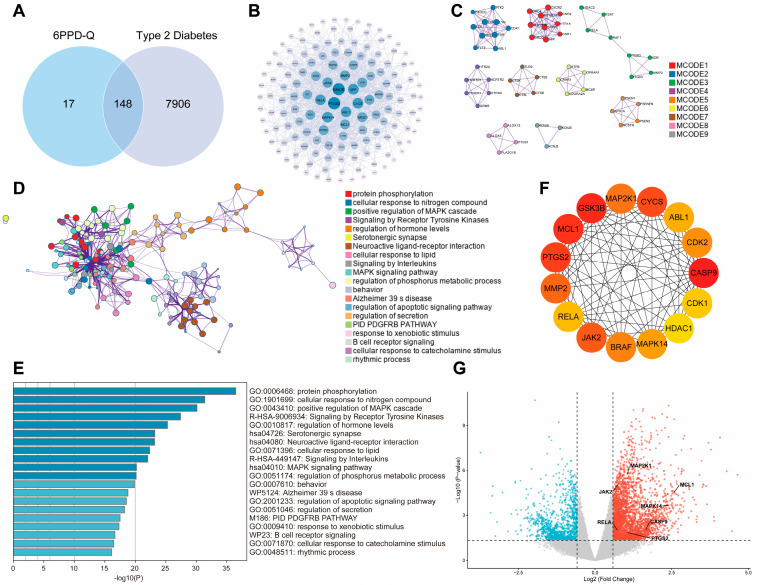
PPI network construction and enrichment analysis of 6PPD-Q-associated targets in type 2 diabetes. (**A**) Venn diagram illustrating the intersection of predicted 6PPD-Q targets and type-2-diabetes-associated genes. (**B**) PPI network of the shared targets constructed via the STRING database (confidence score > 0.40). (**C**) Key functional modules extracted from the PPI network using the MCODE algorithm. (**D**) Network visualization of the enriched GO and KEGG pathways for the intersecting targets. (**E**) Bar chart displaying the top significantly enriched functional pathways, ranked by −log10 (*p*). (**F**) The top 15 hub genes prioritized using the MCC method. The color gradient indicates the topological essentiality of the nodes. (**G**) Transcriptomic validation of the identified hub genes using the type 2 diabetes clinical dataset GSE29231. The volcano plot highlights the differentially expressed core targets (thresholds: *p* < 0.05 and fold change > 1.5).

In the type 2 diabetes context, intersection analysis identified 148 shared targets between 6PPD-Q-related targets and type-2-diabetes-associated genes. These targets formed the basis for the subsequent PPI network and functional enrichment analyses (Figure 6A,B).

MCODE analysis identified dominant clusters associated with trafficking and processing of endosomal TLR (R-HSA-1679131), arachidonic acid metabolism (hsa00590), and Toll-like receptor cascades (R-HSA-168898) (Figure 6C; Table S2), indicating the involvement of innate immunity, inflammatory lipid mediator metabolism, and Toll-like receptor signaling. GO/KEGG enrichment further highlighted striking enrichment in protein phosphorylation (GO:0006468), cellular response to nitrogen compound (GO:1901699), and positive regulation of MAPK cascade (GO:0043410). These pathways reflect amplified kinase activation and cellular stress signaling that may profoundly disrupt homeostasis in type 2 diabetes. Involvement in the regulation of hormone levels (GO:0010817), cellular response to lipid (GO:0071396), and signaling by interleukins (R-HSA-449147) further corroborates that 6PPD-Q drives diabetes-related tissue injury and complication progression through hormonal dysregulation, lipid imbalance, and increased inflammation (Figure 6D,E).

Hub genes ranked by the MCC algorithm were subsequently validated using the GSE29231 dataset. Among them, CASP9, JAK2, MAP2K1, MAPK14, MCL1, PTGS2, and RELA were significantly differentially expressed and are shown in the volcano plot (Figure 6F,G).

### 2.8. The Effects of 6PPD-Q on NAFLD

**Figure 7:**
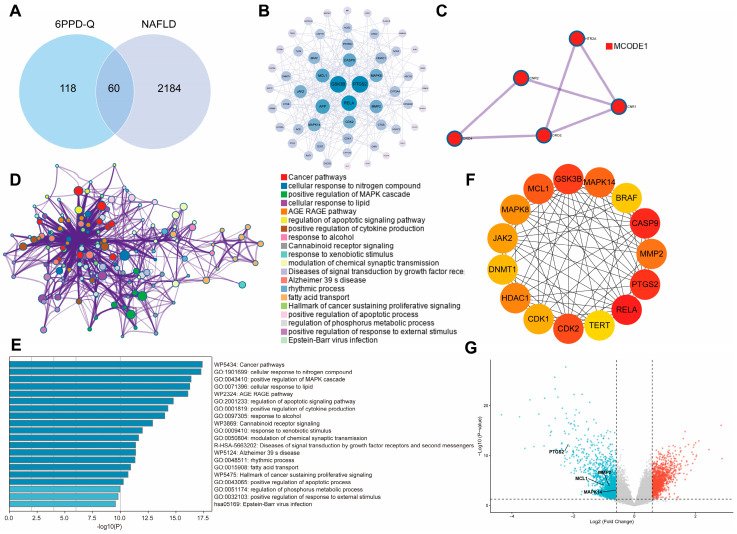
PPI network construction and enrichment analysis of 6PPD-Q-associated targets in NAFLD. (**A**) Venn diagram illustrating the intersection of predicted 6PPD-Q targets and NAFLD-associated genes. (**B**) PPI network of the shared targets constructed via the STRING database (confidence score > 0.40). (**C**) Key functional modules extracted from the PPI network using the MCODE algorithm. (**D**) Network visualization of the enriched GO and KEGG pathways for the intersecting targets. (**E**) Bar chart displaying the top significantly enriched functional pathways, ranked by −log10 (*p*). (**F**) The top 15 hub genes prioritized using the MCC method. The color gradient indicates the topological essentiality of the nodes. (**G**) Transcriptomic validation of the identified hub genes using the NAFLD clinical dataset GSE89632. The volcano plot highlights the differentially expressed core targets (thresholds: *p* < 0.05 and fold change > 1.5).

In the NAFLD context, 60 overlapping targets were obtained between 6PPD-Q-related targets and NAFLD-associated genes. These shared targets were integrated into the PPI network to examine their interaction patterns and functional links (Figure 7A,B).

MCODE analysis showed that 6PPD-Q targets clustered around GPCR class A rhodopsin-like pathways (WP455), G-protein-coupled receptor signaling pathway coupled to cyclic nucleotide second messenger (GO:0007187), and class A/1 rhodopsin-like receptors (R-HSA-373076) (Figure 7C; Table S2). This profile suggests a role for GPCR-mediated signaling and second messenger regulation in 6PPD-Q-induced hepatic injury.

Global enrichment analysis pinpointed the positive regulation of the MAPK cascade (GO:0043410), cellular response to lipid (GO:0071396), and the AGE-RAGE pathway (WP2324). These findings connect 6PPD-Q exposure to severe cellular stress signaling, lipid deposition, and glycation injury. Concurrent enrichment in the regulation of apoptotic signaling pathway (GO:2001233), positive regulation of cytokine production (GO:0001819), and response to xenobiotic stimulus (GO:0009410) implies that 6PPD-Q accelerates NAFLD progression by intensifying hepatocyte apoptosis, robust inflammation, and xenobiotic toxicity (Figure 7D,E).

After MCC-based hub-gene ranking (Figure 7F), hub-gene screening and validation in GSE89632 identified MAPK14, MCL1, MMP2, and PTGS2 as significant core targets (Figure 7G).

### 2.9. Molecular Docking of Core Targets Induced by 6PPD-Q in Atherosclerosis, Type 2 Diabetes, and NAFLD

**Figure 8:**
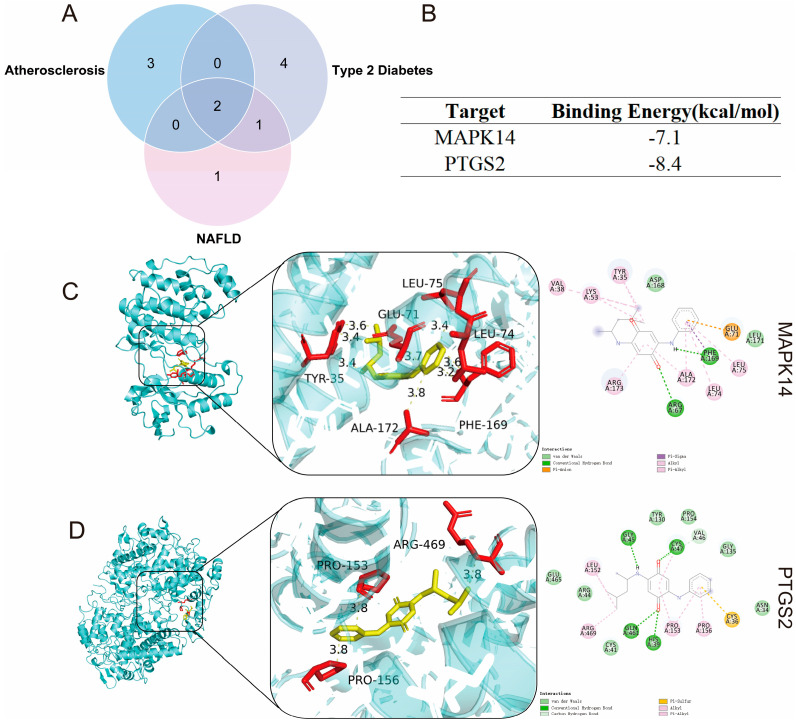
Molecular docking analysis of 6PPD-Q with the shared core targets in lipid-metabolism disorders. (**A**) Venn diagram illustrating the intersection of 6PPD-Q-associated targets with lipid-metabolism disorders, identifying two shared core targets. (**B**) Summary of the binding energies (kcal/mol) between 6PPD-Q and the two shared targets, calculated via AutoDock Vina. (**C**,**D**) Molecular docking simulations demonstrating the optimal binding modes of 6PPD-Q within the active pockets of (**C**) MAPK14 and (**D**) PTGS2. Each panel displays the 3D protein–ligand complex (left), a magnified 3D view highlighting specific interacting amino acid residues and bond distances (middle), and a 2D interaction diagram detailing the types of intermolecular forces, such as hydrogen bonds and hydrophobic contacts (right).

Venn analysis identified MAPK14 and PTGS2 as two shared core targets for 6PPD-Q across atherosclerosis, type 2 diabetes, and NAFLD (Figure 8A). Molecular docking analysis showed favorable binding affinities between 6PPD-Q and these proteins, with binding energies of −7.1 kcal/mol for MAPK14 and −8.4 kcal/mol for PTGS2 (Figure 8B). Among them, PTGS2 showed the lower binding energy, suggesting a more stable predicted interaction with 6PPD-Q.

Detailed examination of the binding conformations showed that 6PPD-Q formed favorable docking poses with MAPK14 and PTGS2, with the ligand positioned within the predicted binding regions of both proteins. The predicted complexes were mainly maintained by hydrogen bonds, hydrophobic contacts, and other non-covalent interactions with neighboring residues (Figure 8C,D). These docking results add structural support to the network-based prioritization of MAPK14 and PTGS2 as key 6PPD-Q-associated nodes across lipid-metabolism-related disease contexts.

### 2.10. Integrative Analysis of the Core Targets for 6PPD and 6PPD-Q

**Figure 9:**
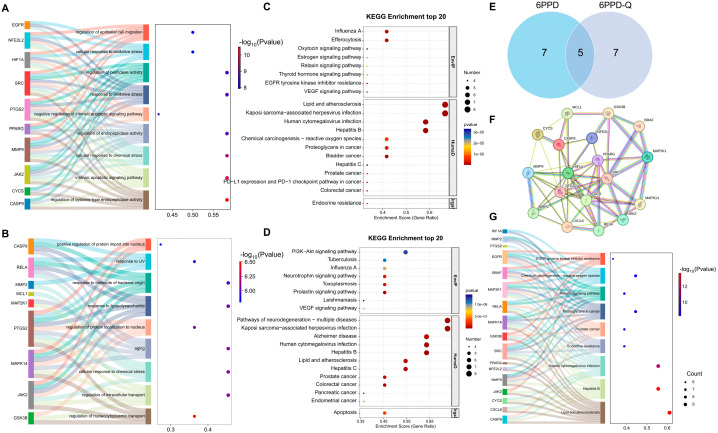
Comprehensive analysis of union core targets of 6PPD and 6PPD-Q. (**A**,**B**) GO analysis of 6PPD (**A**) and 6PPD-Q (**B**) union core targets. (**C**,**D**) KEGG analysis of 6PPD (**C**) and 6PPD-Q (**D**) union core targets. (**E**) Venn diagram showing five common core targets (EGFR, PTGS2, HIF1A, PPARG, SRC) of 6PPD and 6PPD-Q involved in lipid-metabolism disorders, defined as shared core targets. (**F**) PPI network of the combined core targets. (**G**) Sankey diagram illustrating the main KEGG pathways and associated genes for the combined core targets. In all enrichment plots, node size represents gene count, and the color gradient indicates statistical significance −log10 (*p*).

To systematically compare the shared and distinct mechanisms between the parent compound and its transformation product, GO and KEGG enrichment analyses were conducted on their respective core targets across the three diseases. GO analysis revealed that 6PPD-targeted genes were primarily enriched in the regulation of epithelial cell migration, cellular response to oxidative stress, chemical stress, and intrinsic apoptotic signaling pathways, highlighting its pronounced role in oxidative injury and apoptosis. Conversely, 6PPD-Q targets exhibited a shift towards inflammatory stimulus responses (e.g., response to lipopolysaccharide), nucleocytoplasmic transport regulation, and cellular senescence (Figure 9A,B).

KEGG pathway profiling further delineated their divergent modes of action. 6PPD showed distinct enrichment in lipid and atherosclerosis, chemical carcinogenesis-reactive oxygen species, and VEGF signaling, tightly linking it to metabolic derangement and vascular abnormalities. In stark contrast, 6PPD-Q targets were robustly enriched in the PI3K–Akt signaling pathway, neurotrophin signaling, and apoptosis, thereby signaling a deeper involvement in fundamental cell survival and death regulation mechanisms (Figure 9C,D).

Intersection of these core networks identified five universally shared targets between 6PPD and 6PPD-Q (Figure 9E). The integrated PPI network highlighted dense connectivity among these hubs, including EGFR, PTGS2, HIF1A, PPARG, and SRC, which exhibited relatively high topological importance. This suggests that the two compounds may converge on highly interconnected signaling modules rather than isolated targets, thereby providing a mechanistic basis for their coordinated effects on metabolic stress, inflammation, and lipid dysregulation (Figure 9F). Crucially, KEGG analysis of these shared hubs was predominantly enriched for lipid and atherosclerosis, EGFR tyrosine kinase inhibitor resistance, and reactive oxygen species pathways (Figure 9G). Collectively, these integrative findings provide convergent evidence that 6PPD and 6PPD-Q affect overlapping metabolic and inflammatory networks, including MAPK signaling, lipid-inflammation axes, and oxidative-stress-related pathways, which may contribute to lipid-metabolism-related pathological responses. Despite these shared features, the two compounds also appeared to have distinct dominant signatures. 6PPD-associated targets were more closely linked to oxidative stress, lipid and atherosclerosis pathways, CXCL8/MMP9-related inflammatory recruitment, and extracellular matrix remodeling, whereas 6PPD-Q-associated targets showed stronger enrichment in MAPK cascade activation, receptor tyrosine kinase signaling, GPCR-related pathways, and MAPK14/PTGS2-centered stress responses.

### 2.11. In Vivo Experimental Validation of Lipid Metabolism Toxicity of 6PPD and 6PPD-Q

**Figure 10:**
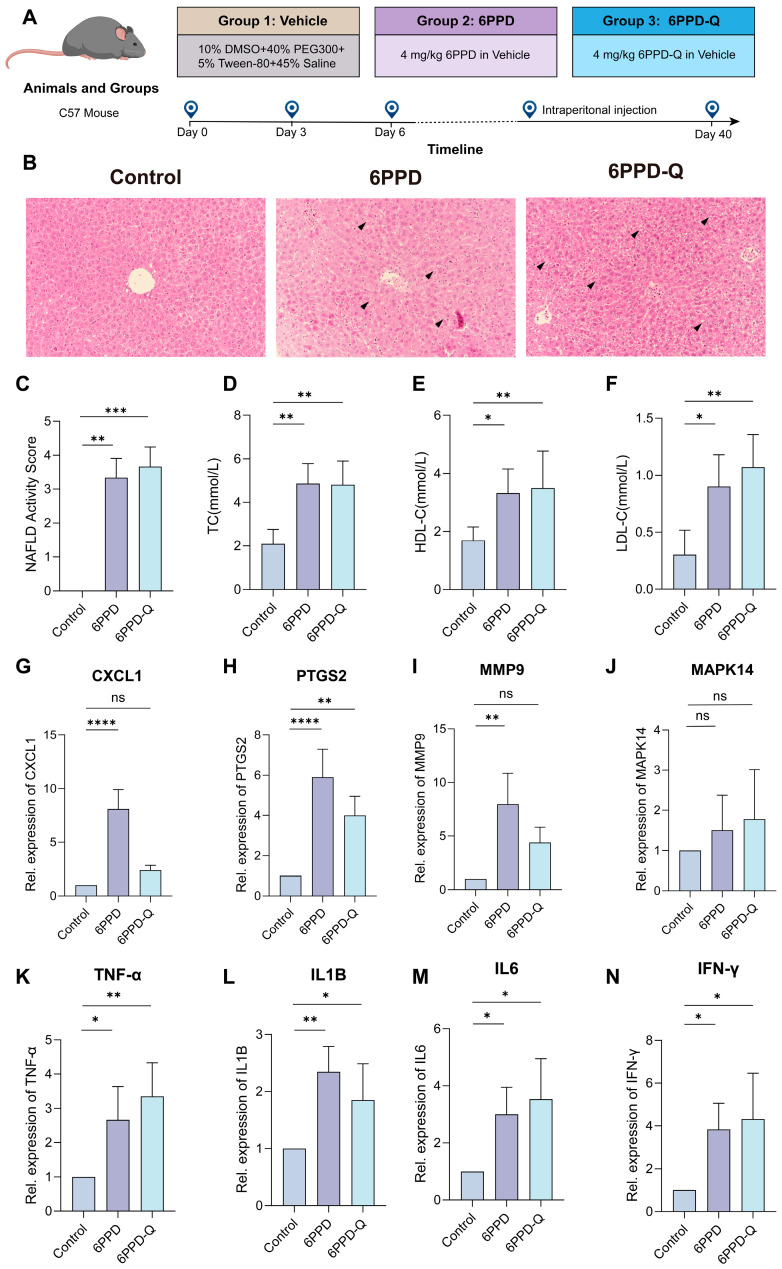
In vivo assessment of metabolic disturbances caused by 6PPD and 6PPD-Q. (**A**) Schematic illustration of the in vivo experimental workflow. C57BL/6 mice in each group were intraperitoneally injected with vehicle, 6PPD (4 mg/kg), or 6PPD-Q (4 mg/kg) every 3 days for 40 days. (**B**) Representative hematoxylin-and-eosin (H&E)-stained images of liver tissues. (**C**) Composite NAFLD activity score derived from blinded semi-quantitative assessment of hepatic steatosis, inflammatory cell infiltration, and hepatocyte ballooning/vacuolar degeneration in H&E-stained liver sections. (**D**–**F**) Quantitative analysis of plasma lipid profiles in mice: (**D**) TC, (**E**) HDL-C, and (**F**) LDL-C. (**G**–**J**) Relative mRNA expression levels of predicted core targets in liver tissues determined by RT-qPCR: (**G**) CXCL1, (**H**) PTGS2, (**I**) MMP9, and (**J**) MAPK14. (**K**–**N**) Relative mRNA expression levels of pro-inflammatory cytokines determined by RT-qPCR: (**K**) TNF-α, (**L**) IL1B, (**M**) IL6, and (**N**) IFN-γ. * *p* < 0.05, ** *p* < 0.01, *** *p* < 0.001, **** *p* < 0.0001.

To validate the predicted metabolic toxicity obtained from computational analysis, we established an in vivo exposure model. C57BL/6 mice received intraperitoneal injections of saline, 6PPD, or 6PPD-Q for 40 days (Figure 10A). Hematoxylin-and-eosin (H&E) staining of liver tissues (Figure 10B) showed that both 6PPD and 6PPD-Q induced varying degrees of hepatic structural disruption, hepatocyte swelling, vacuolar degeneration, and local inflammatory cell infiltration. To further quantify these histopathological alterations, blinded semi-quantitative scoring was performed. Compared with the control group, both 6PPD- and 6PPD-Q-exposed mice showed significantly increased NAFLD activity scores (Figure 10C), indicating aggravated hepatic steatosis, inflammatory infiltration, and hepatocyte ballooning/vacuolar degeneration after pollutant exposure. Plasma biochemical analysis further showed that, compared with the control group, 6PPD and 6PPD-Q significantly increased plasma TC (Figure 10D), HDL-C (Figure 10E), and LDL-C (Figure 10F) levels. These findings indicate that exposure to 6PPD and 6PPD-Q disrupts hepatic metabolic homeostasis and induces metabolic toxicity characterized by hepatocyte injury and lipid-metabolism disorder.

We then used RT-qPCR to assess the mRNA expression of computationally prioritized core targets in liver tissues. Since mice lack a direct CXCL8 ortholog, we assessed CXCL1 expression by RT-qPCR as a murine surrogate marker of CXCL8-related chemokine activity. 6PPD exposure significantly upregulated the expression of CXCL1 (Figure 10G), PTGS2 (Figure 10H), and MMP9 (Figure 10I). 6PPD-Q also markedly increased PTGS2 (Figure 10H) and MMP9 (Figure 10I) expression, whereas MAPK14 showed no obvious change after either treatment (Figure 10J). Because lipid metabolic abnormalities are often linked to chronic inflammation, we further measured the mRNA levels of TNF-α (Figure 10K), IL1B (Figure 10L), IL6 (Figure 10M), and IFNG (Figure 10N). The results showed that all these inflammatory factors were significantly increased in liver tissues after exposure to 6PPD and 6PPD-Q. This further supports the predicted activation of inflammation-related pathways by both compounds. Together, these results suggest that 6PPD and 6PPD-Q may aggravate metabolic toxicity by activating hepatic inflammation and further disturbing lipid metabolic homeostasis.

### 2.12. In Vitro Experimental Validation of 6PPD- and 6PPD-Q-Mediated Cytotoxicity, Oxidative Stress, and Inflammatory Activation

**Figure 11:**
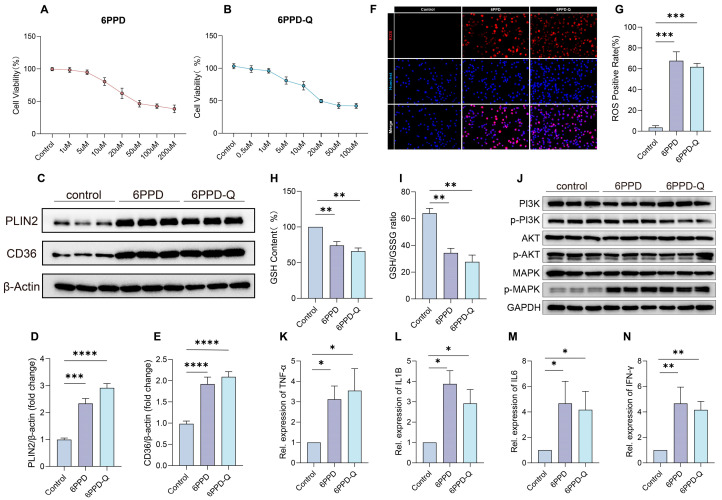
6PPD and 6PPD-Q induce lipid-accumulation-related protein expression, oxidative stress, MAPK signaling activation, and pro-inflammatory responses in RAW 264.7 macrophages. (**A**) Cell viability of RAW 264.7 cells following exposure to increasing concentrations of 6PPD. (**B**) Cell viability of RAW 264.7 cells following exposure to increasing concentrations of 6PPD-Q. (**C**) Representative Western blot images showing the protein expression levels of PLIN2 and CD36 after treatment with 6PPD or 6PPD-Q. β-Actin was used as the loading control. (**D**,**E**) Densitometric analysis of PLIN2 (**D**) and CD36 (**E**) protein expression normalized to β-actin and expressed relative to the control group. (**F**) Representative fluorescence images showing intracellular ROS accumulation. ROS-positive signals are shown in red, and nuclei stained with Hoechst are shown in blue. (**G**) Quantification of ROS-positive cells. (**H**,**I**) Intracellular glutathione redox markers, including GSH content (**H**) and the GSH/GSSG ratio (**I**), after 6PPD or 6PPD-Q exposure. (**J**) Representative Western blot images showing PI3K, p-PI3K, AKT, p-AKT, MAPK, and p-MAPK protein levels after 6PPD or 6PPD-Q exposure. GAPDH was used as the loading control. (**K**–**N**) Relative mRNA expression levels of pro-inflammatory cytokines determined by RT-qPCR, including TNF-α (**K**), IL1B (**L**), IL6 (**M**), and IFN-γ (**N**). Data are presented as mean ± SD from three independent biological replicates. Statistical significance was determined by one-way ANOVA followed by Tukey’s multiple-comparison test. * *p* < 0.05, ** *p* < 0.01, *** *p* < 0.001, **** *p* < 0.0001.

To further validate the predicted lipid metabolic and inflammatory effects of 6PPD and 6PPD-Q, RAW 264.7 macrophages were used for in vitro experiments. Cell viability assays showed that both compounds reduced cell viability in a concentration-dependent manner, indicating cytotoxic effects after exposure (Figure 11A,B). Consistent with lipid-metabolism disturbance, Western blot analysis showed increased protein expression of PLIN2 and CD36 in both the 6PPD- and 6PPD-Q-treated groups, and densitometric analysis confirmed the upregulation of these lipid-accumulation-related markers (Figure 11C–E). We next examined oxidative stress and redox homeostasis. ROS fluorescence staining showed markedly enhanced ROS signals after 6PPD or 6PPD-Q exposure, and quantification confirmed a significant increase in ROS-positive cells (Figure 11F,G). Meanwhile, intracellular GSH content and the GSH/GSSG ratio, calculated based on GSH and GSSG measurements, were both reduced, indicating disruption of cellular redox homeostasis (Figure 11H,I). To provide protein-level support for the predicted signaling pathways, PI3K–AKT and MAPK signaling were further examined by Western blotting. Under the present experimental conditions, p-PI3K and p-AKT levels did not show obvious changes, whereas p-MAPK band intensity was increased after exposure to either compound, suggesting activation of MAPK signaling in RAW 264.7 macrophages (Figure 11J). Finally, RT-qPCR analysis showed that 6PPD and 6PPD-Q significantly upregulated TNF-α, IL1B, IL6, and IFN-γ expression (Figure 11K–N). Together, these findings indicate that 6PPD and 6PPD-Q induce cytotoxicity, lipid-accumulation-related protein dysregulation, oxidative stress, glutathione redox imbalance, MAPK phosphorylation, and inflammatory activation in RAW 264.7 macrophages.

## 3. Discussion

The ubiquitous presence of tire-derived pollutants, particularly the antioxidant 6PPD and its ozonation product 6PPD-Q, has become an important concern in environmental toxicology and public health research [20]. Since 6PPD-Q was identified as a key toxicant responsible for acute mortality in coho salmon exposed to urban stormwater runoff, research on this compound class has rapidly expanded from aquatic lethality to mammalian toxicity, human exposure, and long-term health risk assessment [4]. Recent studies have detected 6PPD, 6PPD-Q, and related p-phenylenediamine quinones in human biological samples, indicating that these chemicals are not only environmental contaminants but also potential internal exposure hazards [21,22,23]. However, compared with ecological toxicity and acute organ injury, the metabolic consequences of 6PPD and 6PPD-Q exposure remain insufficiently defined. This gap is especially important because lipid-metabolism disorders, oxidative stress, and chronic inflammation are central mechanisms linking environmental exposure to atherosclerosis, type 2 diabetes, and NAFLD [24].

The present study addresses this knowledge gap by integrating network toxicology, transcriptomic validation, molecular docking, and experimental verification. By analyzing atherosclerosis, type 2 diabetes, and NAFLD together, and by validating selected predictions in mouse liver tissues and RAW 264.7 macrophages, this study links predicted molecular targets with biological outcomes. This integrated approach provides a clearer view of how tire-derived chemicals may contribute to lipid-metabolism-related toxicity through coordinated effects on lipid dysregulation, inflammatory activation, oxidative stress, tissue remodeling, and cellular stress responses.

Our computational analysis identified EGFR, PTGS2, HIF1A, PPARG, and SRC as shared core targets of 6PPD and 6PPD-Q across lipid-metabolism-related diseases. These molecules represent key regulatory nodes in lipid handling, inflammatory signaling, hypoxic stress, insulin sensitivity, and kinase-mediated cellular adaptation. Among them, PTGS2 is particularly notable. PTGS2 is involved in arachidonic acid metabolism and eicosanoid production, both of which are closely linked to vascular inflammation, insulin resistance, and hepatic inflammatory injury [25]. In the present study, PTGS2 was identified not only as a shared computational target and a stable docking partner but also as upregulated in liver tissues after exposure. This consistency between network prediction, molecular docking, and experimental validation suggests that PTGS2-centered inflammatory signaling may be a key bridge between 6PPD/6PPD-Q exposure and lipid metabolic injury.

The in vivo validation further strengthens the biological relevance of our computational findings. The liver is a central organ for xenobiotic metabolism and lipid homeostasis, and it is also one of the major target organs for metabolic toxicants. In our mouse model, repeated exposure to 6PPD or 6PPD-Q caused hepatic structural injury, including hepatocyte swelling, vacuolar degeneration, and inflammatory cell infiltration. These histological changes were accompanied by marked alterations in circulating lipid parameters, including increased TC, HDL-C, and LDL-C levels. Together, these findings indicate that both compounds can disturb hepatic metabolic homeostasis and alter systemic lipid profiles at the whole-organism level. This experimental evidence supports our computational prediction that 6PPD and 6PPD-Q are closely associated with lipid response, inflammatory regulation, and hepatic metabolic injury. Moreover, this observation is consistent with recent evidence showing that low-dose 6PPD-Q exposure can induce fatty liver-like changes in mice by promoting fatty acid biosynthesis [13]. It also aligns with multi-omics studies suggesting that 6PPD and 6PPD-Q may promote NAFLD-related changes by disturbing gut–liver metabolic homeostasis [14].

The inflammatory changes observed in liver tissues provide another important layer of evidence. Chronic low-grade inflammation is a common pathological driver of NAFLD, type 2 diabetes, and atherosclerosis [26]. In the present study, both 6PPD and 6PPD-Q increased the hepatic expression of TNF-α, IL1B, IL6, and IFN-γ. These cytokines can impair lipid metabolism, promote insulin resistance, enhance endothelial dysfunction, and accelerate the transition from simple lipid accumulation to more severe inflammatory tissue injury. The upregulation of CXCL8 and MMP9 also supports a role for immune cell recruitment and extracellular matrix remodeling. In the context of atherosclerosis, CXCL8 and MMP9 are closely associated with leukocyte chemotaxis, vascular wall inflammation, matrix degradation, and plaque instability [27,28]. Thus, the hepatic qPCR results not only validate selected predicted targets but also support a broader mechanism in which 6PPD and 6PPD-Q promote metabolic toxicity through inflammation-associated tissue remodeling.

The in vitro experiments further support these findings at the cellular level. Macrophages are key effector cells in metabolic inflammation and participate in NAFLD, atherosclerosis, and insulin resistance. In RAW 264.7 macrophages, both 6PPD and 6PPD-Q reduced cell viability, increased intracellular ROS accumulation, and enhanced pro-inflammatory cytokine expression. These results suggest that macrophages are responsive to 6PPD and 6PPD-Q exposure. They are also consistent with previous reports that 6PPD-Q induces cytotoxicity, ROS production, and inflammatory responses in liver and lung cell models [29,30]. The additional Western blot results provide protein-level support for the cellular pathway responses predicted by network toxicology. In RAW 264.7 macrophages, 6PPD and 6PPD-Q exposure did not obviously alter total PI3K, AKT, or MAPK protein levels. However, increased p-MAPK band intensity was observed after exposure to either compound, which is consistent with the enrichment of MAPK-related signaling in the network analysis. Together with the cellular stress and cytokine results, this finding provides protein-level support for the MAPK-related signaling response predicted by the network analysis.

A central implication of this study is that 6PPD and 6PPD-Q should not be considered toxicologically equivalent. Although they share several disease-related pathways, their dominant toxicological signatures are different. 6PPD was more closely associated with oxidative stress, CXCL8/MMP9-related inflammatory recruitment, and tissue remodeling. In contrast, 6PPD-Q showed stronger links with MAPK-related signaling, PTGS2/MAPK14-centered networks, inflammatory stimulus response, and broader cellular stress pathways. This distinction is supported by recent liver cell data showing that 6PPD-Q has lower IC50 values than 6PPD, suggesting higher toxicity toward human liver cells [29]. Therefore, environmental transformation should not be viewed as a simple chemical conversion. Instead, it may generate products with altered biological activity and distinct toxic mechanisms. This finding supports the need to evaluate 6PPD-Q and other PPD-quinones independently in environmental risk assessment.

These findings also have disease-specific implications. In NAFLD, hepatic injury, dyslipidemia, ROS accumulation, and inflammatory activation may promote progression from early metabolic stress to inflammatory liver damage [31]. In type 2 diabetes, inflammatory cytokines and oxidative stress may worsen insulin resistance and disrupt glucose–lipid metabolic coupling [32]. In atherosclerosis, macrophage activation, LDL-related lipid stress, and matrix remodeling may contribute to vascular inflammation and plaque progression [33]. Recent human evidence showing associations between serum 6PPD-Q levels, glycolipid metabolic disturbance, immune dysregulation, and liver lesion risk further supports the potential relevance of these mechanisms to lipid-metabolism-related health outcomes [8].

From a broader environmental health perspective, this study adds to the growing view that traffic-related pollutants should be assessed not only by their inhalation toxicity or aquatic lethality, but also by their potential to disturb systemic metabolism. Tire wear particles and their chemical additives are continuously released into road dust, airborne particles, soil, and runoff. Human exposure may occur through inhalation, ingestion, and contact with contaminated environmental media. Because metabolic diseases develop through long-term, low-grade biological stress, even moderate pollutant-induced changes in lipid metabolism, oxidative stress, and inflammation may have public health significance. Therefore, our findings support a shift in future monitoring strategies from measuring only environmental occurrence to evaluating metabolic and immune-related health endpoints.

From an environmental and public health perspective, our findings suggest that tire-derived contaminants, such as 6PPD and 6PPD-Q, may be relevant to metabolic health risk assessment, particularly through their potential involvement in oxidative stress, inflammatory activation, and lipid dysregulation. Given the widespread use of 6PPD in tire rubber and the environmental occurrence of its transformation product 6PPD-Q, further studies are warranted to evaluate realistic exposure levels, chronic low-dose effects, combined exposures, and susceptible populations. Environmental monitoring, toxicological validation, and epidemiological investigations will be important for clarifying the broader health implications of these pollutants and for supporting future risk assessment and pollution control strategies.

Several limitations should be acknowledged. First, network toxicology and molecular docking are predictive and hypothesis-generating approaches. They are useful for integrating compound-target, disease-gene, and pathway information and for prioritizing potential mechanisms, but they cannot independently prove direct target binding, target activation/inhibition, or causal disease-related effects. Second, although our in vivo and in vitro experiments provide biological validation, the animal exposure route was intraperitoneal injection, which does not fully mimic common human exposure routes such as inhalation and ingestion. Third, the current experimental validation focused mainly on histology, plasma lipid parameters, mRNA expression, ROS production, and cell viability. Protein-level validation, phosphorylation assays, pathway inhibition experiments, Oil Red O staining, hepatic triglyceride measurement, and lipidomics are needed to further define the mechanism. Fourth, RAW 264.7 cells are useful for macrophage-based inflammatory studies but cannot fully represent liver-resident macrophages, vascular macrophages, or multi-organ metabolic crosstalk. Finally, disease-specific models, such as high-fat-diet-induced NAFLD, diabetic mouse models, and ApoE^−/−^ atherosclerosis models, will be needed to determine whether 6PPD and 6PPD-Q directly accelerate disease initiation or progression.

Overall, this study provides a comprehensive mechanistic framework linking 6PPD and 6PPD-Q exposure to lipid-metabolism-related toxicity. By integrating multi-disease network analysis, molecular docking, mouse experiments, and macrophage validation, our work demonstrates that these tire-derived pollutants may disturb metabolic homeostasis through coordinated effects on hepatic injury, oxidative stress, inflammatory activation, and lipid dysregulation. These findings expand the toxicological understanding of 6PPD and 6PPD-Q from environmental occurrence and acute toxicity toward chronic metabolic health risk and provide a theoretical and experimental basis for future mechanistic studies, biomarker discovery, and environmental health risk assessment.

## 4. Materials and Methods

### 4.1. Toxicological Analysis of 6PPD and 6PPD-Q

Toxicity predictions for 6PPD and 6PPD-Q were conducted using the ADMETlab 3.0 platform to evaluate their potential toxicological properties. The canonical SMILES strings for both compounds were sourced from the PubChem database.

### 4.2. Compilation of Targets for 6PPD and 6PPD-Q

To initiate target prediction, the canonical SMILES strings of 6PPD and 6PPD-Q were retrieved from the PubChem database and queried against four target prediction or compound–protein interaction platforms: SwissTargetPrediction (https://swisstargetprediction.ch/), TargetNet (http://targetnet.scbdd.com/), ChEMBL (www.ebi.ac.uk/chembl/), and STITCH (https://stitch-db.org/) [34,35,36,37]. All searches were restricted to Homo sapiens to ensure human relevance. For SwissTargetPrediction, predicted targets with a probability score > 0 were retained. For TargetNet, targets with a probability score ≥ 0.5 were included. For ChEMBL, curated human protein targets with a target confidence score ≥ 6 were retained. For STITCH, compound–protein interactions with a combined confidence score ≥ 0.40 were included.

The predicted targets obtained from these databases were then merged and standardized. Protein identifiers, gene names, and aliases were converted into official Homo sapiens gene symbols using the UniProtKB IDMapping service. Duplicate entries were removed based on official gene symbols. Non-human targets, unmapped entries, ambiguous annotations, and repeated gene symbols were excluded. When the same target was identified by multiple databases, it was retained only once in the final candidate target list.

### 4.3. Identification of Target Networks Associated with Lipid-Metabolism-Related Diseases

To explore the potential mechanisms linking 6PPD and 6PPD-Q to lipid-metabolism-related disorders, this study focused on three representative diseases: atherosclerosis, type 2 diabetes, and NAFLD. Disease-associated targets for these three conditions were independently retrieved from the GeneCards database. GeneCards was selected because it integrates disease–gene associations from multiple genomic, transcriptomic, proteomic, functional annotation, and literature-based resources, making it suitable for broad disease-target collection in network toxicology studies. For each disease term, genes with a GeneCards relevance score > 5 were retained to preserve broad coverage of disease-associated molecular networks.

The retrieved disease targets were standardized into official Homo sapiens gene symbols using the UniProtKB IDMapping service. Duplicate entries, unmapped targets, and ambiguous annotations were removed. The target datasets for atherosclerosis, type 2 diabetes, and NAFLD were analyzed separately to preserve disease-specific pathological contexts. Venn diagrams were then used to identify intersecting targets between the compound-associated target sets and each disease-associated gene set. These overlapping genes were designated as potential compound–disease intersection targets for subsequent PPI network construction, hub target screening, and enrichment analysis.

### 4.4. Construction of Protein Interaction Network and Screening of Hub Targets

To elucidate the molecular interactions and biological pathways mediating 6PPD- and 6PPD-Q-induced lipid-metabolism disorders, we independently constructed Protein–Protein Interaction (PPI) networks for the intersecting target sets of atherosclerosis, type 2 diabetes, and NAFLD using the STRING database (http://string-db.org). For these networks, the organism was restricted to Homo sapiens, and a minimum required interaction score of 0.40 was applied. The constructed networks were visualized and analyzed in Cytoscape (version 3.10.3), where key topological features, including degree centrality, closeness centrality, and betweenness centrality, were assessed. Core potential targets for atherosclerosis, type 2 diabetes, and NAFLD were identified using the cytoHubba plug-in, applying the maximal clique centrality (MCC) algorithm. MCC was selected due to its proven ability to identify biologically meaningful hub genes by scoring nodes based on their involvement in multiple maximum cliques, outperforming simpler metrics in complex biological networks.

### 4.5. Analysis of Target Protein Function and Pathway Enrichment

Functional enrichment analysis was performed for the distinct target sets of atherosclerosis, type 2 diabetes, and NAFLD using Metascape (version 3.5). This integrated Gene Ontology (GO) classification and Kyoto Encyclopedia of Genes and Genomes (KEGG) pathway analysis, with pathways considered statistically significant at *p* < 0.05 and a minimum gene overlap of three. Furthermore, the molecular complex detection (MCODE) algorithm was applied to detect densely connected network modules, which often represent critical protein complexes linked to lipid-metabolism disorders and metabolic inflammation. The findings were visualized using network diagrams and bubble plots, highlighting the specific metabolic pathways and molecular alterations associated with 6PPD and 6PPD-Q exposure in the context of atherosclerosis, type 2 diabetes, and NAFLD.

### 4.6. Screening of Key Targets for Lipid Metabolism Disorders via Transcriptomic Validation

To enhance the predictive accuracy of core targets associated with lipid-metabolism disorders, the Gene Expression Omnibus (GEO) database was utilized to download clinical transcriptomic datasets related to atherosclerosis, type 2 diabetes, and NAFLD. Specifically, the datasets GSE66360, GSE29231, and GSE89632 were selected for atherosclerosis [38], type 2 diabetes [39], and NAFLD [40], respectively. Detailed characteristics of these three datasets are provided in Supplementary Table S3. The expression profiles of potential targets for 6PPD and 6PPD-Q were independently analyzed within these specific disease cohorts. The criteria for screening differentially expressed genes (DEGs) were strictly defined as *p* < 0.05 and |fold change (FC)|≥ 1.5. By intersecting these DEGs with our previously established network targets for atherosclerosis, type 2 diabetes, and NAFLD, we identified the potential functional targets for 6PPD and 6PPD-Q, designating them as the core targets mediating lipid-metabolism disorders. Finally, the ggplot2 package in R(version 4.5.2) was employed to construct volcano plots, distinctively annotating these core targets to facilitate the visual interpretation of their expression patterns.

### 4.7. Molecular Docking

Molecular docking was performed using AutoDock Vina (version 1.1.2) to evaluate the binding affinities between the target compounds (6PPD and 6PPD-Q) and the identified core targets. Protein preparation, encompassing the separation of native ligands, removal of water molecules, and deletion of heteroatoms, was conducted using PyMOL (version 4.3.0). Subsequently, AutoDockTools(version 1.5.7) was employed to add polar hydrogen atoms, calculate Gasteiger charges, and define the docking grid boxes and rotatable bonds. Both the ligand and receptor structures were converted into the PDBQT format for semi-flexible docking simulations. Post-docking protein–ligand interactions were visualized in 3D using PyMOL and in 2D using Discovery Studio Visualizer. A binding energy of <0 kcal/mol indicates spontaneous binding, whereas a value of ≤−5.0 kcal/mol signifies a highly stable and strong binding interaction.

### 4.8. In Vivo Exposure Model

All animal handling and experimental protocols were reviewed and approved by the Institutional Animal Care and Use Committee of Central South University. The study utilized 8-week-old healthy male C57BL/6 mice, which were randomly allocated into three distinct groups: vehicle control, 6PPD exposure (4 mg/kg), and 6PPD-Q exposure (4 mg/kg). Over a 40-day period, the corresponding solutions were intraperitoneally injected into the mice once every three days. The body weight of each animal was documented prior to every injection to monitor their growth and health status. At the end of the 40-day regimen, profound anesthesia was induced in mice using 1% sodium pentobarbital (70 mg/kg). Following the verification of deep anesthesia through the loss of the pedal withdrawal reflex, blood samples were drawn, and liver tissue was rapidly excised for subsequent biochemical and molecular assays.

### 4.9. Histology

Liver tissues were harvested and fixed in 4% paraformaldehyde (PFA), followed by paraffin embedding. Serial sections were deparaffinized, rehydrated through a graded ethanol series, and stained with hematoxylin and eosin (H&E) using routine procedures. Histopathological changes were examined, and images were acquired using a Nikon ECLIPSE Ci light microscope (Tokyo, Japan) coupled with a Nikon DS-U3 digital camera. Histopathological scoring was performed by two independent investigators blinded to the treatment groups. H&E-stained liver sections were semi-quantitatively evaluated for steatosis, inflammatory cell infiltration, and hepatocyte ballooning/vacuolar degeneration using criteria adapted from the NAFLD Activity Score system and previous mouse NASH studies. Steatosis was scored as 0 (<5%), 1 (5–33%), 2 (34–66%), or 3 (>66%); inflammatory infiltration was scored as 0 (none), 1 (<2 foci/200× field), 2 (2–4 foci/200× field), or 3 (>4 foci/200× field); hepatocyte ballooning/vacuolar degeneration was scored as 0 (none), 1 (mild/few affected cells), or 2 (moderate-to-severe/many affected cells). The component scores for steatosis, inflammatory infiltration, and hepatocyte ballooning/vacuolar degeneration were summed to generate a composite NAFLD activity score for each animal, which was used for statistical analysis.

### 4.10. Serum Lipid Analysis

Blood samples were collected from mice and centrifuged at 3000 rpm for 15 min to obtain serum. Serum lipid parameters, including total cholesterol (TC), low-density lipoprotein cholesterol (LDL-C), and high-density lipoprotein cholesterol (HDL-C), were measured automatically using an automated biochemical analyzer (Hitachi 7180, Tokyo, Japan) with matching commercial reagents.

### 4.11. Cell Culture

RAW264.7 cells were cultured in Dulbecco’s Modified Eagle Medium (DMEM) supplemented with 10% fetal bovine serum, 1% penicillin/streptomycin (100 U/mL penicillin and 100 μg/mL streptomycin) at 37 °C in a humidified incubator containing 5% CO_2_. The medium was refreshed every 2 days, and cells were passaged upon reaching 70–80% confluence.

### 4.12. Cell Viability Assessment

To assess the viability of RAW264.7 cells, cells were seeded into 96-well plates at a density of 4 × 10^3^ cells per well and allowed to adhere overnight. The cells were then treated with varying concentrations of 6PPD or 6PPD-Q for 48 h. After the exposure period, cell viability was measured using the Cell Counting Kit-8 (CCK-8) assay (Beyotime Biotechnology, Shanghai, China). Briefly, CCK-8 solution was added to each well, and the plates were incubated at 37 °C for an appropriate time. Absorbance was finally measured at 450 nm using a microplate reader.

### 4.13. Western Blot

Total proteins from RAW 264.7 cells were extracted using RIPA buffer containing protease and phosphatase inhibitors (Roche, Basel, Switzerland) and quantified using a BCA assay (Thermo Fisher Scientific, Waltham, MA, USA). Equal amounts of protein (20 μg/lane) were separated by SDS-PAGE and transferred onto PVDF membranes (Millipore, Burlington, MA, USA). After blocking, the membranes were incubated overnight at 4 °C with primary antibodies against PLIN2, CD36, PI3K, p-PI3K, AKT, p-AKT, MAPK, p-MAPK, β-actin, or GAPDH. After washing with TBST, the membranes were incubated with HRP-conjugated secondary antibodies for 1 h at room temperature. Protein bands were visualized using an ECL detection system and quantified using ImageJ (version 1.53) software. The expression levels of PLIN2 and CD36 were normalized to β-actin.

### 4.14. Quantitative Real-Time PCR (RT-qPCR)

Total RNA was extracted from treated RAW264.7 cells using the FastPure^®^ Cell/Tissue Total RNA Isolation Kit (Vazyme, Nanjing, China). Reverse transcription was performed with the TransScript All-in-One First-Strand cDNA Synthesis SuperMix (TransGen, Beijing, China) to generate cDNA. Quantitative PCR amplification was subsequently carried out on a Roche LightCycler 480 system using the TransScript Probe One-Step qRT-PCR SuperMix (TransGen, China). Relative gene expression levels were normalized to the housekeeping gene β-actin, and fold changes were calculated using the 2^−ΔΔCT^ method.

### 4.15. Intracellular ROS Detection

Intracellular ROS production was assessed in treated RAW264.7 cells using the CellROX^®^ Red Reagent (Invitrogen, Carlsbad, CA, USA). Briefly, after exposure to the compounds, cells were washed twice with pre-warmed PBS and incubated with 1 μM CellROX^®^ Red probe in serum-free DMEM at 37 °C for 30 min under light-protected conditions. Unbound probe was removed by three PBS washes. Nuclear staining was performed with Hoechst 33342 (1 μg/mL) for 5 min. Fluorescent images were acquired using a fluorescence microscope, where red signals represented intracellular ROS levels.

### 4.16. Intracellular GSH and GSSG Measurement

Intracellular GSH and GSSG levels were measured using a GSH and GSSG Assay Kit (Beyotime, S0053, China) according to the manufacturer’s instructions. Briefly, after treatment with 6PPD or 6PPD-Q, RAW 264.7 cells were washed with PBS and collected by centrifugation. The cell pellets were resuspended in protein removal reagent M, vortexed thoroughly, and subjected to two rapid freeze–thaw cycles using liquid nitrogen and a 37 °C water bath. After incubation on ice for 5 min, the samples were centrifuged at 10,000× *g* for 10 min at 4 °C, and the supernatants were collected for glutathione detection.

For total glutathione measurement, samples or standards were mixed with total glutathione detection working solution containing DTNB and glutathione reductase, followed by addition of NADPH. Absorbance at 412 nm was measured using a microplate reader. For GSSG measurement, endogenous GSH was removed using the GSH scavenging auxiliary solution and GSH scavenging reagent before detection. GSH content was calculated as total glutathione minus two times GSSG, and the GSH/GSSG ratio was calculated as an indicator of intracellular glutathione redox balance.

### 4.17. Statistical Analysis

Quantitative results from at least three independent experiments are expressed as the mean ± SD. All statistical analyses were performed using GraphPad Prism version 10.1.2. For comparisons between two groups, a two-tailed unpaired Student’s *t*-test was applied. Differences among multiple groups were analyzed by one-way ANOVA with post hoc multiple comparisons. A *p*-value < 0.05 was considered statistically significant (* *p* < 0.05, ** *p* < 0.01, *** *p* < 0.001, **** *p* < 0.0001; ns = not significant).

## 5. Conclusions

This study elucidates a potential mechanistic framework by which the tire-derived pollutant 6PPD and its environmental transformation product 6PPD-Q contribute to lipid-metabolism-related diseases, including atherosclerosis, type 2 diabetes, and NAFLD. By integrating network toxicology, transcriptomic validation, molecular docking, and experimental verification, we identified shared and compound-specific toxicological features. EGFR, PTGS2, HIF1A, PPARG, and SRC were identified as shared core targets, suggesting that both pollutants converge on lipid dysregulation, oxidative stress, inflammatory signaling, and metabolic injury. The in vivo and in vitro experiments further strengthened these predictions. In C57BL/6 mice, repeated exposure to 6PPD and 6PPD-Q caused hepatic steatosis, altered circulating lipid profiles, and activated inflammatory cytokines and predicted core targets in liver tissues. In vitro experiments, both compounds reduced cell viability, promoted ROS accumulation, and enhanced pro-inflammatory cytokine expression. These results indicate that 6PPD and 6PPD-Q can induce metabolic toxicity through hepatic injury, oxidative stress, macrophage inflammatory activation, and lipid metabolic disturbance. Importantly, 6PPD and 6PPD-Q showed partially distinct toxicological signatures, highlighting the need to evaluate environmental transformation products independently from their parent compounds. Overall, our findings provide mechanistic and experimental evidence that tire-derived pollutants may represent an underrecognized environmental metabolic risk, supporting the need for improved monitoring, source control, and safer alternatives to 6PPD-based tire antioxidants.

## Figures and Tables

**Table 1:** Predicted probabilities of toxicity for 6PPD and 6PPD-Q.

Chemicals	Toxicity	Probability
6PPD	DILI	0.920
	Skin Sensitization	0.901
	Respiratory	0.864
	Human Hepatotoxicity	0.788
	Drug-induced Nephrotoxicity	0.709
	Drug-induced Neurotoxicity	0.695
	Hematotoxicity	0.400
	Genotoxicity	0.966
6PPD-Q	DILI	0.858
	Skin Sensitization	0.975
	Respiratory	0.611
	Human Hepatotoxicity	0.830
	Drug-induced Nephrotoxicity	0.597
	Drug-induced Neurotoxicity	0.570
	Hematotoxicity	0.740
	Genotoxicity	0.994

## Data Availability

The data presented in this study are available on request from the corresponding author. The transcriptomic datasets analyzed during the current study are available in the GEO repository, under accession numbers GSE66360, GSE29231, and GSE89632.

## Supplementary Materials

The following supporting information can be downloaded at https://www.mdpi.com/article/10.3390/ijms27156712/s1.

## Abbreviations

The following abbreviations are used in this manuscript:

6PPD	N-(1,3-dimethylbutyl)-N’-phenyl-p-phenylenediamine
6PPD-Q	6PPD-quinone
NAFLD	Nonalcoholic fatty liver disease
DILI	Drug-induced liver injury
PPI	Protein–protein interaction
GO	Gene ontology
KEGG	Kyoto Encyclopedia of Genes and Genomes
MCC	Maximal clique centrality
SMILES	Simplified molecular-input line-entry system
GEO	Gene Expression Omnibus
MCODE	Molecular Complex Detection
ROS	Reactive oxygen species

## References

[1] Jiang Y., Wang C., Ma L., Gao T., Wang Y. (2024). Environmental profiles, hazard identification, and toxicological hallmarks of emerging tire rubber-related contaminants 6PPD and 6PPD-quinone. Environ. Int..

[2] Cao G., Wang W., Zhang J., Wu P., Zhao X., Yang Z., Hu D., Cai Z. (2022). New Evidence of Rubber-Derived Quinones in Water, Air, and Soil. Environ. Sci. Technol..

[3] Yi J., Ruan J., Yu H., Wu B., Zhao J., Wang H., Chen R., Yang Q., Chen J., Sun D. (2025). Environmental fate, toxicity, and mitigation of 6PPD and 6PPD-Quinone: Current understanding and future directions. Environ. Pollut..

[4] Tian Z., Zhao H., Peter K.T., Gonzalez M., Wetzel J., Wu C., Hu X., Prat J., Mudrock E., Hettinger R. (2021). A ubiquitous tire rubber-derived chemical induces acute mortality in coho salmon. Science.

[5] Liu C., Zhao X., Guo L., Yu Q., Zhang W., Peng Z., Gao Y., Gong X., Li P., Jiao H. (2024). Emerging N-(1,3-dimethylbutyl)-N’-phenyl-p-phenylenediamine (6PPD) and 6PPD quinone in paired human plasma and urine from Tianjin, China: Preliminary assessment with demographic factors. J. Hazard. Mater..

[6] Wu X., Hu J., Yuan Z., Wang S., Tong L. (2024). p-phenylenediamines (PPDs) and PPD-quinones (PPD-Qs) in human urine and breast milk samples: Urgent need for focus on PPD-Qs and the establishment of health threshold criteria. J. Hazard. Mater..

[7] Zhang J., Liu H., Zhao H., Dai Q., Wang W., Wang F., Cao G., Zhou Y., Xu S., Cai Z. (2025). Patterns, variability, and predictors of p-phenylenediamine quinones and parent p-phenylenediamines in pregnant women across three trimesters. Environ. Int..

[8] Qin Z., Li Y., Qin Y., Chen Z., Guo J., Fang F., Schaffer A., Hollert H., Shao Y. (2025). Correlation between 6PPD-Q and immune along with metabolic dysregulation induced liver lesions in outdoor workers. Environ. Int..

[9] Weinberg Sibony R., Segev O., Dor S., Raz I. (2024). Overview of oxidative stress and inflammation in diabetes. J. Diabetes.

[10] El-Sehrawy A., Mohammed A.N., Gupta J., Mohammed J.S., Roopashree R., Kashyap A., Janney J.B., Sahoo S., Al-Hasnaawei S., Nasr Y.M. (2025). Combating oxidative stress in non-alcoholic fatty liver disease: From mechanisms to therapeutic strategies. Pathol. Res. Pract..

[11] Kopych V., Da Costa A.D.S., Park K. (2025). Endothelial Dysfunction in Atherosclerosis: Experimental Models and Therapeutics. Biomater. Res..

[12] Wang Y., Wu J., Wang D. (2025). 6-PPD quinone causes lipid accumulation across multiple generations differentially affected by metabolic sensors and components of COMPASS complex in Caenorhabditis elegans. Environ. Pollut..

[13] Wang L., Tang W., Sun N., Lv J., Hu J., Tao L., Zhang C., Wang H., Chen L., Xu D.X. (2025). Low-dose tire wear chemical 6PPD-Q exposure elicit fatty liver via promoting fatty acid biosynthesis in ICR mice. J. Hazard. Mater..

[14] Feng Y., Xian H., Bai R., Li Z., Li H., Zhang L., Huang X., Huang Y., Liang B., Deng Y. (2025). Multi-omics insights into 6PPD- and 6PPDQ-induced gut-liver axis disruption and non-alcoholic fatty liver disease progression in zebrafish (*Danio rerio*). J. Hazard. Mater..

[15] Wang L., Fu Y., Xu S., Chen E., Zhang S., Yang D., He Y., Gu Y., Mao Y., Yuan F. (2025). Targeting the PI3K-AKT pathway to alleviate 6PPDQ-induced cognitive impairment associated with metabolic syndrome. Ecotoxicol. Environ. Saf..

[16] Greer J.B., Dalsky E.M., Lane R.F., Hansen J.D. (2023). Tire-Derived Transformation Product 6PPD-Quinone Induces Mortality and Transcriptionally Disrupts Vascular Permeability Pathways in Developing Coho Salmon. Environ. Sci. Technol..

[17] Lv M., Mao X., Lu Z., Yang Y., Huang J., Cheng Y., Ye C., He Z., Shu L., Mo D. (2025). 6PPD induces cerebrovascular defects by triggering oxidative stress and ferroptosis in zebrafish. Sci. Total Environ..

[18] Huang S. (2023). Efficient analysis of toxicity and mechanisms of environmental pollutants with network toxicology and molecular docking strategy: Acetyl tributyl citrate as an example. Sci. Total Environ..

[19] Norouzi S., Nahmiach N., Perez G., Zhu Y., Peslherbe G.H., Muir D.C.G., Zhang X. (2025). Molecular docking for screening chemicals of environmental health concern: Insight from a case study on bisphenols. Environ. Sci. Process Impacts.

[20] Zhang S., Tang J., Qiu Z., Huo X., Liu D., Zeng X. (2025). Environmental and Human Health Risks of 6PPD and 6PPDQ: Assessment and Implications. Toxics.

[21] Du B., Liang B., Li Y., Shen M., Liu L.-Y., Zeng L. (2022). First Report on the Occurrence of N-(1,3-Dimethylbutyl)-N′-phenyl-p-phenylenediamine (6PPD) and 6PPD-Quinone as Pervasive Pollutants in Human Urine from South China. Environ. Sci. Technol. Lett..

[22] Mao W., Jin H., Guo R., Chen P., Zhong S., Wu X. (2024). Occurrence of p-phenylenediamine antioxidants in human urine. Sci. Total Environ..

[23] Deng M., Ji X., Peng B., Fang M. (2024). In Vitro and In Vivo Biotransformation Profiling of 6PPD-Quinone toward Their Detection in Human Urine. Environ. Sci. Technol..

[24] Sule R.O., Rivera G.D.T., Vaidya T., Gartrell E., Gomes A.V. (2025). Environmental Toxins and Oxidative Stress: The Link to Cardiovascular Diseases. Antioxidants.

[25] Wang B., Wu L., Chen J., Dong L., Chen C., Wen Z., Hu J., Fleming I., Wang D.W. (2021). Metabolism pathways of arachidonic acids: Mechanisms and potential therapeutic targets. Signal Transduct. Target. Ther..

[26] Caussy C., Aubin A., Loomba R. (2021). The Relationship Between Type 2 Diabetes, NAFLD, and Cardiovascular Risk. Curr. Diab Rep..

[27] Di Nubila A., Dilella G., Simone R., Barbieri S.S. (2024). Vascular Extracellular Matrix in Atherosclerosis. Int. J. Mol. Sci..

[28] Zernecke A., Weber C. (2010). Chemokines in the vascular inflammatory response of atherosclerosis. Cardiovasc. Res..

[29] Qi Y., Qiu A., Wei X., Huang Y., Huang Q., Huang W. (2024). Effects of 6PPD-Quinone on Human Liver Cell Lines as Revealed with Cell Viability Assay and Metabolomics Analysis. Toxics.

[30] Damdam S., Hunnie B., Jones P., Hecker M., Brinkmann M., da Silva Junior F.C. (2026). Toxicity assessment of 6PPD-quinone in human lung cells: Insights from BEAS-2B and A549 cell lines. Toxicol. Vitr..

[31] Chen Z., Tian R., She Z., Cai J., Li H. (2020). Role of oxidative stress in the pathogenesis of nonalcoholic fatty liver disease. Free Radic. Biol. Med..

[32] Rehman K., Akash M.S. (2016). Mechanisms of inflammatory responses and development of insulin resistance: How are they interlinked?. J. Biomed. Sci..

[33] Libby P. (2002). Inflammation in atherosclerosis. Nature.

[34] Kuhn M., Szklarczyk D., Pletscher-Frankild S., Blicher T.H., von Mering C., Jensen L.J., Bork P. (2014). STITCH 4: Integration of protein-chemical interactions with user data. Nucleic Acids Res..

[35] Daina A., Michielin O., Zoete V. (2019). SwissTargetPrediction: Updated data and new features for efficient prediction of protein targets of small molecules. Nucleic Acids Res..

[36] Yao Z.J., Dong J., Che Y.J., Zhu M.F., Wen M., Wang N.N., Wang S., Lu A.P., Cao D.S. (2016). TargetNet: A web service for predicting potential drug-target interaction profiling via multi-target SAR models. J. Comput. Aided Mol. Des..

[37] Zdrazil B., Felix E., Hunter F., Manners E.J., Blackshaw J., Corbett S., de Veij M., Ioannidis H., Lopez D.M., Mosquera J.F. (2024). The ChEMBL Database in 2023: A drug discovery platform spanning multiple bioactivity data types and time periods. Nucleic Acids Res..

[38] Muse E.D., Kramer E.R., Wang H., Barrett P., Parviz F., Novotny M.A., Lasken R.S., Jatkoe T.A., Oliveira G., Peng H. (2017). A Whole Blood Molecular Signature for Acute Myocardial Infarction. Sci. Rep..

[39] Jain P., Vig S., Datta M., Jindel D., Mathur A.K., Mathur S.K., Sharma A. (2013). Systems biology approach reveals genome to phenome correlation in type 2 diabetes. PLoS ONE.

[40] Arendt B.M., Comelli E.M., Ma D.W., Lou W., Teterina A., Kim T., Fung S.K., Wong D.K., McGilvray I., Fischer S.E. (2015). Altered hepatic gene expression in nonalcoholic fatty liver disease is associated with lower hepatic n-3 and n-6 polyunsaturated fatty acids. Hepatology.

