# Supplementary material for: Unveiling the Molecular Mechanism of 6PPD and 6PPD-Q in Lipid-Metabolism-Related Diseases Through Network Toxicology and Experimental Validation
Source: Int J Mol Sci. 2026 Jul 27;27(15):6712. doi: 10.3390/ijms27156712 (PMC13467288; doi:10.3390/ijms27156712)
Supplement: Supplementary file 1 [file ijms-27-06712-s001.zip › ijms-4416894-supplementary.pdf]

Table S1. Top 3 terms of MCODE analysis for 6PPD targets

Diseases	MCODE	GO	Description	Log10(P)
Atherosclerosis	MCODE1	WP2586	Aryl hydrocarbon receptor pathway	-14.9
		WP5144	NRP1 triggered signaling in pancreatic cancer	-14.4
		hsa05200	Pathways in cancer	-13.9
	MCODE2	hsa04726	Serotonergic synapse	-15.7
		R-HSA-2142753	Arachidonate metabolism	-15.3
		GO:0019369	arachidonate metabolic process	-15.2
	MCODE3	R-HSA-1679131	Trafficking and processing of endosomal TLR	-14.5
		GO:0030574	collagen catabolic process	-11.8
		R-HSA-1474228	Degradation of the extracellular matrix	-11.4
	MCODE4	R-HSA-418594	G alpha (i) signalling events	-17.8
		R-HSA-373076	Class A/1 (Rhodopsin-like receptors)	-17.6
		R-HSA-500792	GPCR ligand binding	-16.3
	MCODE5	M44	PID HIF2PATHWAY	-7.9
		GO:0071363	cellular response to growth factor stimulus	-6.5
		GO:0070848	response to growth factor	-6.4
Type 2 diabetes	MCODE1	R-HSA-193692	Regulated proteolysis of p75NTR	-16.5
		R-HSA-205043	NRIF signals cell death from the nucleus	-15.1
		WP5434	Cancer pathways	-14.7
	MCODE2	WP5470	Effect of omega 3 PUFA on Huntington 39 s disease pathways	-16.1
		WP4829	mBDNF and proBDNF regulation of GABA neurotransmission	-15.3
		GO:1904862	inhibitory synapse assembly	-13.8
	MCODE3	GO:0006690	icosanoid metabolic process	-17.1
		hsa00590	Arachidonic acid metabolism	-17
		hsa04726	Serotonergic synapse	-14.8
	MCODE4	R-HSA-418594	G alpha (i) signalling events	-25.8
		R-HSA-388396	GPCR downstream signalling	-21.9
		R-HSA-372790	Signaling by GPCR	-21.2
	MCODE5	R-HSA-180786	Extension of Telomeres	-6.1
		WP5144	NRP1 triggered signaling in pancreatic cancer	-6.1
		GO:0090398	cellular senescence	-5.9
	MCODE6	R-HSA-416476	G alpha (q) signalling events	-10.8
		hsa04080	Neuroactive ligand-receptor interaction	-9.6
		R-HSA-500792	GPCR ligand binding	-9.1
	MCODE7	R-HSA-418555	G alpha (s) signalling events	-9.2
		GO:0007189	adenylate cyclase-activating G protein-coupled receptor signaling pathway	-8.9
		GO:0007188	adenylate cyclase-modulating G protein-coupled receptor signaling pathway	-8.2
	MCODE8	R-HSA-1296059	G protein gated Potassium channels	-8.5
		R-HSA-997272	Inhibition of voltage gated Ca2+ channels via Gbeta/gamma subunits	-8.5
		R-HSA-1296041	Activation of G protein gated Potassium channels	-8.5

NAFLD	MCODE1	WP5434	Cancer pathways	-12.7
		hsa05200	Pathways in cancer	-12.5
		GO:0141124	intracellular signaling cassette	-11
	MCODE2	R-HSA-1679131	Trafficking and processing of endosomal TLR	-8.1
		GO:0032760	positive regulation of tumor necrosis factor production	-7.2
		GO:1903557	positive regulation of tumor necrosis factor superfamily cytokine production	-7.2
	MCODE3	WP2586	Aryl hydrocarbon receptor pathway	-13.4
		WP5113	Antiviral and anti inflammatory effects of Nrf2 on SARS CoV 2 pathway	-10.8
		R-HSA-9818749	Regulation of NFE2L2 gene expression	-9.6
	MCODE4	hsa00591	Linoleic acid metabolism	-15.2
		GO:0016098	monoterpenoid metabolic process	-14.7
		WP5324	Octadecanoid formation from linoleic acid	-13
	MCODE5	GO:0030162	regulation of proteolysis	-5

Table S2. Top 3 terms of MCODE analysis for 6PPD-Q targets

Diseases	MCODE	GO	Description	Log10(P)
Atherosclerosis	MCODE1	WP5434	Cancer pathways	-16.7
		hsa05200	Pathways in cancer	-16.5
		hsa05165	Human papillomavirus infection	-13.5
	MCODE2	R-HSA-418594	G alpha (i) signalling events	-11.9
		WP247	Small ligand GPCRs	-11.8
		R-HSA-373076	Class A/1 (Rhodopsin-like receptors)	-11.7
	MCODE3	R-HSA-1679131	Trafficking and processing of endosomal TLR	-17.2
		R-HSA-168898	Toll-like Receptor Cascades	-11.3
		GO:0030574	collagen catabolic process	-10.8
	MCODE4	R-HSA-9017802	Noncanonical activation of NOTCH3	-14.7
		GO:0034205	amyloid-beta formation	-14.4
		GO:0007220	Notch receptor processing	-14.4
	MCODE5	WP3932	Focal adhesion PI3K Akt mTOR signaling	-8
		R-HSA-9656223	Signaling by RAF1 mutants	-8
		R-HSA-6802955	Paradoxical activation of RAF signaling by kinase inactive BRAF	-7.9
	MCODE6	WP167	Eicosanoid synthesis	-9.4
		WP5432	Omega 3 fatty acids in senescence	-9
		WP5122	Prostaglandin and leukotriene metabolism in senescence	-8.9
Type 2 diabetes	MCODE1	R-HSA-1679131	Trafficking and processing of endosomal TLR	-14.1
		hsa00590	Arachidonic acid metabolism	-10.4
		R-HSA-168898	Toll-like Receptor Cascades	-10.3
	MCODE2	R-HSA-373076	Class A/1 (Rhodopsin-like receptors)	-19.6
		R-HSA-500792	GPCR ligand binding	-18.1
		R-HSA-372790	Signaling by GPCR	-16.3
	MCODE3	GO:0038094	Fc-gamma receptor signaling pathway	-18.7
		GO:0038093	Fc receptor signaling pathway	-17.1
		GO:0038096	Fc-gamma receptor signaling pathway involved in phagocytosis	-16.7
	MCODE4	WP5530	MAPK signaling and ARTD family members	-12.6
		hsa05161	Hepatitis B	-11.3
		WP2032	Thyroid stimulating hormone TSH signaling	-11.3
	MCODE5	GO:0007200	phospholipase C-activating G protein-coupled receptor signaling pathway	-13.9
		R-HSA-416476	G alpha (q) signalling events	-12.9
		hsa04080	Neuroactive ligand-receptor interaction	-11.5
	MCODE6	R-HSA-9017802	Noncanonical activation of NOTCH3	-18.6
		GO:0034205	amyloid-beta formation	-18.2
		GO:0007220	Notch receptor processing	-18.2
	MCODE7	R-HSA-418555	G alpha (s) signalling events	-11.5
		GO:0007189	adenylate cyclase-activating G protein-coupled receptor signaling pathway	-11.2
		GO:0007188	adenylate cyclase-modulating G protein-coupled receptor signaling pathway	-10.2

	MCODE8	M63	PID AVB3 OPN PATHWAY	-9
		GO:0001704	formation of primary germ layer	-7.2
		hsa05418	Fluid shear stress and atherosclerosis	-7
	MCODE9	R-HSA-997272	Inhibition of voltage gated Ca2+ channels via Gbeta/gamma subunits	-9.1
		R-HSA-1296041	Activation of G protein gated Potassium channels	-9.1
		R-HSA-1296059	G protein gated Potassium channels	-9.1
NAFLD	MCODE1	WP455	GPCRs class A rhodopsin like	-10.3
		GO:0007187	G protein-coupled receptor signaling pathway, coupled to cyclic nucleotide second messenger	-10.3
		R-HSA-373076	Class A/1 (Rhodopsin-like receptors)	-9.8

Table S3. Sources and analysis of GEO datasets related to lipid metabolism disorders.

Disease	Dataset	Experimental Design Parameters	Source of Biological Samples
Atherosclerosis	GSE66360	This research involved the isolation of circulating endothelial cells from whole blood samples of patients experiencing acute myocardial infarction (n=49) and healthy cohorts (n=50). Subsequently, gene expression patterns of the isolated cells were measured and analyzed using the HG-133U_PLUS_2 microarray platform.	*Homo sapiens*, endothelial cells
Type 2 diabetes	GSE29231	This research involved the collection of biopsy samples of visceral adipose tissue from female patients with type 2 diabetes (n=3) and non-diabetic female cohorts (n=3). To generate robust expression profiles, three biological replicates with four technical replicates each were utilized. Subsequently, gene expression patterns of the samples were measured and analyzed using the Illumina HumanHT-12 v3 Expression BeadChip microarray platform.	*Homo sapiens*, visceral adipose tissue
NAFLD	GSE89632	This research involved the collection of hepatic tissue samples from patients with non-alcoholic fatty liver disease (n=39) and healthy cohorts (n=24). Subsequently, gene expression patterns of the samples were measured and analyzed using the Illumina microarray platform.	*Homo sapiens*, hepatic tissue
